# MK-801 Impairs Cognitive Coordination on a Rotating Arena (Carousel) and Contextual Specificity of Hippocampal Immediate-Early Gene Expression in a Rat Model of Psychosis

**DOI:** 10.3389/fnbeh.2014.00075

**Published:** 2014-03-12

**Authors:** Štěpán Kubík, Helena Buchtová, Karel Valeš, Aleš Stuchlík

**Affiliations:** ^1^Neurophysiology of Memory, Institute of Physiology, Academy of Sciences of the Czech Republic, Prague, Czech Republic

**Keywords:** hippocampus, arc, homer 1a, place avoidance, rotating arena, carousel, cognitive coordination, schizophrenia

## Abstract

Flexible behavior in dynamic, real-world environments requires more than static spatial learning and memory. Discordant and unstable cues must be organized in coherent subsets to give rise to meaningful spatial representations. We model this form of cognitive coordination on a rotating arena – Carousel where arena- and room-bound spatial cues are dissociated. Hippocampal neuronal ensemble activity can repeatedly switch between multiple representations of such an environment. Injection of tetrodotoxin into one hippocampus prevents cognitive coordination during avoidance of a stationary room-defined place on the Carousel and increases coactivity of previously unrelated neurons in the uninjected hippocampus. Place avoidance on the Carousel is impaired after systemic administration of non-competitive NMDAr blockers (MK-801) used to model schizophrenia in animals and people. We tested if this effect is due to cognitive disorganization or other effect of NMDAr antagonism such as hyperlocomotion, spatial memory impairment, or general learning deficit. We also examined if the same dose of MK-801 alters patterns of immediate-early gene (IEG) expression in the hippocampus. IEG expression is triggered in neuronal nuclei in a context-specific manner after behavioral exploration and it is used to map activity in neuronal populations. IEG expression is critical for maintenance of synaptic plasticity and memory consolidation. We show that the same dose of MK-801 that impairs spatial coordination of rats on the Carousel also eliminates contextual specificity of IEG expression in hippocampal CA1 ensembles. This effect is due to increased similarity between ensembles activated in different environments, consistent with the idea that it is caused by increased coactivity between neurons, which did not previously fire together. Our data support the proposition of the Hypersynchrony theory that cognitive disorganization in psychosis is due to increased coactivity between unrelated neurons.

## Introduction

**Figure 1:**
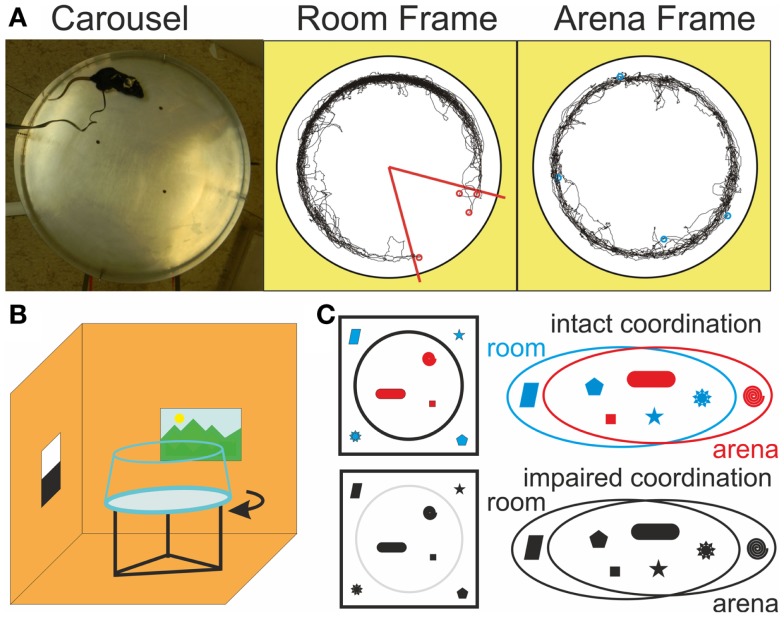
**Dissociated reference frames on the Carousel**. **(A)** A photograph of the Carousel arena (left) and example trajectories in the stationary Room frame (middle), where the shock zone (red) is defined and in the rotating Arena frame (right) during a room-defined place avoidance session. Red and blue circles mark locations where shocks were administered in the Room and Arena frames, respectively. **(B)** Schematics of the rotating arena in a stationary room. Continuous rotation of the arena dissociates spatial reference frames of the stationary room and the rotating arena **(C)** Illustration of the cognitive coordination function in a color model, where cue color facilitates discrimination between two sets of cues. When color is lost, discrimination becomes more difficult. The two cue sets are still clearly delineated from the top view (left), but the distinction is much less clear from the perspective of the animal, let alone a multi-modal spatial representation system in the hippocampus (right).

Dynamic environments in the real world often involve multiple reference frames without stable mutual relationships. Drivers need to keep control of their cars and attend to the road at the same time. Similarly, animals need to keep track of their positions within a group of conspecifics as well as within ambient environment. Elements of experience must be segregated into coherent, meaningful representations to support context-relevant behavioral responses (Figures 1A,C). Impairment of this cognitive coordination could be responsible for cognitive disorganization in psychosis including inappropriate associations, deficits in contextual binding, and impaired discrimination between relevant and irrelevant information (Ellenbroek and Cools, 1990; Silverstein et al., 2000; Phillips and Silverstein, 2003; Uhlhaas et al., 2004; Hemsley, 2005). Spatial, contextual, and episodic-like memory critically depends on the hippocampus (Moscovitch et al., 2006) and hippocampal pathology (Harrison, 2004; Tamminga et al., 2012; Ledoux et al., 2013) and episodic memory deficits are common in schizophrenia (Weiss et al., 2003; Barch, 2005; Boyer et al., 2007; Ranganath et al., 2008). However, most laboratory tests of hippocampal function occur in static environments or involve only one-off changes or between-session alternations.

**Figure 2:**
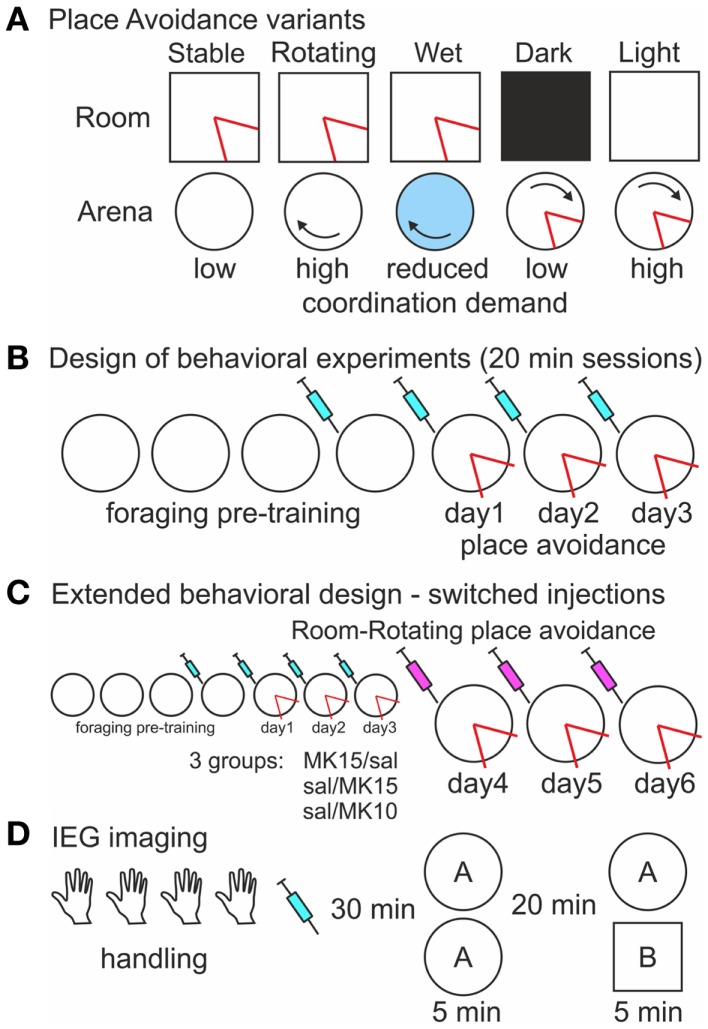
**Experimental design (A)**. A schematic of five versions of the place avoidance task. All spatial cues are relevant and the demand for cognitive coordination is low during room-defined avoidance on the Stable arena when the arena rotation is off. In contrast, the room and arena cues are dissociated on the Rotating arena resulting in a high coordination demand. Cues on the arena surface are hidden by shallow water, but the arena-bound idiothetic cues can still interfere with the avoidance, and the demand for coordination is reduced on the Wet arena. The cognitive coordination demand during arena-based avoidance is low when irrelevant room cues are hidden by Darkness and high when they are available in Light. The shock zone (red) is defined in one reference frame **(B)**. Design of the place avoidance experiments. The rats were pre-trained to forage on the arena prior to the place avoidance training. Saline or MK-801 was administered 30 min before the session starting from the last day of foraging **(C)**. Extended place avoidance training design. After 3 days of Room-Rotating training, injections were switched so that rats initially trained with MK-801 (0.15 mg/kg) received saline (MK15/sal) and rats initially trained with saline received MK-801 at 0.15 mg/kg (sal/MK15) or 0.10 mg/kg (sal/MK10). The extended training continued for 3 days. **(D)**. Design of the IEG imaging experiment. Rats received four handling sessions (5 min), an injection of saline or MK-801, and two exploration sessions in environment **(A)** or in environments**(A,B)**.

During place avoidance (PA) on a continuously rotating circular arena (Carousel), rats have to avoid a place hidden in either the stationary room or the rotating arena frame (Figures 1A,B; Cimadevilla et al., 2001). They have to selectively attend to cues from the relevant frame and ignore the others to avoid the shock effectively. Inactivation of one hippocampus by tetrodotoxin (TTX) increased coactivity of previously uncorrelated CA1 neurons in the uninjected hippocampus (Olypher et al., 2006) and impaired cognitive coordination on the Carousel (Wesierska et al., 2005). PA on the Carousel is also impaired after systemic administration of dizocilpine (MK-801; Stuchlík et al., 2004; Stuchlík and Valeš, 2005; Valeš et al., 2006; Bubeníková-Valešová et al., 2008b). Non-competitive NMDA receptor antagonists such as MK-801 are used to model schizophrenia in animals (Deutsch et al., 2002; Powel and Geyer, 2007; Bubeníková-Valešová et al., 2008a; Adell et al., 2012), because they elicit psychosis in people (Newcomer and Krystal, 2001). We tested if the PA deficit on the Carousel is due to MK-801-induced cognitive disorganization or another effect of NMDAr antagonism such as hyperlocomotion (Martin et al., 1997) or spatial navigation and memory deficits (Stuchlík et al., 2004; van der Staay et al., 2011). We compared the effect of MK-801 on PA in five versions of the task, which differed in the amount of misleading information from the irrelevant spatial frame left to interfere with the PA (Figure 2A).

Behavioral activity triggers immediate-early gene (IEGs) expression in hippocampal and cortical neurons (Kubik et al., 2007, 2012). Precise temporal regulation of transcription and intracellular distribution of RNA for IEG *Arc/Arg3.1* (Link et al., 1995; Lyford et al., 1995) has been used to map activity history of hippocampal pyramidal neurons during two distinct behavioral epochs (Guzowski et al., 1999). Intranuclear foci expression of *Arc* marked neurons activated immediately before the sacrifice, whereas cytoplasmic signal marked neurons activated ~25 min earlier. Although the IEGs *Arc* and *Homer1a* are co-expressed in the same neurons, the signal from riboprobes targeting the 3′ UTR of the long (~40 kb) primary transcript of *Homer 1a* only appears in the nuclei ~25 min after the induction and can be used to replace the diffuse cytoplasmic *Arc* signal (Vazdarjanova et al., 2002).

Proportions of hippocampal neurons expressing *Arc* or *Homer 1a* after exploration of an environment (Guzowski et al., 1999; Vazdarjanova and Guzowski, 2004) are similar to proportions of neurons displaying place cell activity in an environment (Lee et al., 2004; Leutgeb et al., 2004). Both IEG expression and place cell activity occur in an environment-specific manner suggesting that IEG expression is triggered in place cells displaying firing fields during behavioral exploration. The Hypersynchrony theory posits that increased coactivity selectively between previously non-coactive neurons disrupts cognitive coordination and causes cognitive disorganization in psychosis (Fenton, 2009). Neurons active in the same environment are more likely to be coactive than neurons active in different environments. Ensembles of hippocampal neurons expressing *Arc* and *Homer 1a* are more similar after repeated exploration of the same environment than after exploration of different environments (Guzowski et al., 1999; Vazdarjanova and Guzowski, 2004). We tested if MK-801 increases similarity between hippocampal ensembles expressing the IEGs in different environments more than between ensembles activated in the same environment.

## Materials and Methods

### Subjects

One hundred and fifty-one young adult (~3 month old) male Long–Evans rats from the Institute of Physiology breeding colony were used in the study. They were housed in pairs in transparent Plexiglas cages and maintained at a 12/12 h light/dark cycle with lights on at 7:00 and water available *ad libitum*. The rats were acclimatized to the laboratory vivarium for 10 days, handled, and habituated to the experimental apparatus prior to the PA training. This design was adopted to reduce learning-related stress later during training. After the conclusion of behavioral procedures, rats were killed by an overdose of Thiopental. Rats used in the IEG imaging experiment (*n* = 42) were housed individually in opaque cages to avoid high IEG expression background resulting from social interactions. The animals were handled for 4 days, but not habituated to the environment prior to behavioral sessions used to trigger IEG expression (see below). All behavioral testing was conducted during the light phase of the day. All animal treatment complied with the Czech Animal Protection Act, EU directive 2010/63/EC, and NIH guidelines.

### Drug treatment

MK-801 [dizocilpine (+)-5-methyl-10,11-dihydro-5*H*-dibenzo cycloheptene-5,10-imine maleate; Sigma] was diluted in sterile physiological saline at a *low* (0.10 mg/kg) or *high* (0.15 mg/kg) dose. Beginning with the last foraging session, rats received daily injections of either MK-801 or saline solution (1 ml/kg, i.p.) 30 min before behavioral testing (Figures 2B,C). The first injection allowed rats to habituate to the injection procedure and to the MK-801-induced state before the onset of the PA training. Rats in the IEG imaging experiment received a single injection 30 min prior to the catFISH test sessions (Figure 2D).

### Carousel

The apparatus was a smooth metallic elevated circular arena (82 cm diameter) equipped with a 5 cm high lip on the perimeter, a detachable transparent plexiglass wall, and a motor, which rotated the arena (1 rpm; Figure 1B). The wall consisted of four parts connected by small bolts arranged in four vertical strips and prevented the rats from accidentally or deliberately falling off the arena. This symmetrical design was chosen because it provided less spatial information than asymmetrical single joint strip. The wall was always in the same position relative to the arena surface and as such provided arena-based visual information. The rats wore a harness carrying infrared (IR) LEDs and an alligator clip for shock delivery. A tracking system (iTrack, Biosignal Group, Inc., New York, USA) recorded the rat’s position via an overhead IR camera, delivered shocks to reinforce the PA behavior, and stored the data for off-line analysis. The harness was attached to a cable carrying power for the LEDs and the shock current. AC shock current (50 Hz, 0.5 s, 0.5–0.7 mA) was delivered from a constant-current source via the alligator clip connected to a subcutaneously implanted electrode between the rat’s shoulders. It was made by piercing the rat’s skin by a sterile hypodermic needle and twisting the sharp end to prevent slipping out. This design was adopted to facilitate perception of the shock at the high-impedance (~100 kΩ) contact between the rats body and the grounded arena floor rather than at the low-impedance (~100 Ω) implanted electrode. A second IR LED was fixed to the arena perimeter and used by the tracking system to compute a virtual “arena frame” view of the rotating arena (Figure 1A). Indirect light was provided by a 40 W light bulb during all behavioral sessions.

### Place avoidance

After food-restriction to 90% of their pre-restriction weight, rats were trained in four foraging sessions with no shocks and subsequently given three PA training sessions on seven consecutive days (Figure 2B). Some rats received extended PA training on three more days (Figure 2C). Each session lasted 20 min. Cereal cocoa puffs (Nesquik; Nestlé) were dispersed from an overhead feeder to ensure sufficient exploration throughout the experiment and prevent passivity. Importantly, the cocoa puffs remained floating and did not dissolve (Kubik and Fenton, 2005) when the arena was covered with shallow water in one of the experiments (see below). The rats were weighed and their weights recorded daily to maintain constant motivation. During the PA training, a computer-based tracking system (Tracker, Biosignal Group, USA) administered a mild 500 ms shock and counted an entrance whenever the rat entered a 60° shock zone for at least 500 ms. Additional shocks were delivered every 1500 ms until the rat left the shock zone. Additional entrances were counted if the rat left the area for at least 1500 ms. To adjust for variability in shock sensitivity, shock intensity was set individually for each rat to the lowest value sufficient to elicit an escape response, but not freezing. This adjustment was performed on the arena during initial PA training. This design was adopted to enhance comparability between different versions of the PA task by stimulating exploration and discouraging passivity.

### Experimental design

One hundred and nine rats were trained in five different variants of the PA task (Figure 2A). Two doses (0.10 and 0.15 mg/kg) of MK-801 were used in the first two experiments. Thirty-one rats (16 saline, 8 low, and 7 high dose of MK-801) were trained to avoid a place defined in the stationary room on a stable (Room-Stable) and 29 rats (15 saline, 7 low, and 7 high dose of MK-801) on a rotating (Room-Rotating) dry arena. The low dose showed no effect on the rotating arena and therefore only the high dose was used in the following experiments. Sixteen rats (eight saline and eight high MK-801) were tested on the rotating arena covered with shallow water (<1 cm; Room-Wet). Twenty-eight rats were trained to avoid a place defined on the rotating arena either in darkness (Arena-Dark; 12 rats; 6 saline and 6 high MK-801) or in the light (Arena-Light; 16 rats; 8 saline and 8 high MK-801). Conditions during foraging pre-training always matched those during avoidance training except that no shocks were delivered. In the Room-Rotating version, the same PA training was *Extended* for three more days (Figure 2C), but the injections were switched so that rats initially trained with MK-801 (0.15 mg/kg) received saline (MK15/sal, *n* = 7) and rats trained with saline received either 0.15 mg/kg (sal/MK15, *n* = 7) or 0.10 mg/kg MK-801 (sal/MK10, *n* = 7). Rats in the sal/MK10 group showed asymptotic performance from PA day 3 with no MK-801-induced impairment on day 4 and they were used as controls in this experiment. One of the rats did not complete the extended training and was not included in the analysis.

### Behavioral data analysis

Stored data files were analyzed by Track Analysis (Biosignal Group, Inc.). Data from the 3 days of PA training are reported (Figure 2B). The total number of Entrances (*E*) into the shock zone, the Maximum Avoidance Time (MaxT), and the proportion of Time spent in the shock zone (pTime) per session were used to evaluate the avoidance. The total Distance (*D*) traveled during a session measured the overall locomotion. To assess the effect of increased locomotion on the avoidance, the number of Entrances per unit Distance was calculated (ED). In experiments on the rotating arena, we obtained a measure of purely active locomotion (without the arena rotation) in a virtual “arena view” calculated by the tracking system using the reference IR LED fixed to the arena. A two-way ANOVA with repeated measures on sessions was used to compare the effect of MK-801 to vehicle controls over multiple days of training in each condition and to examine the effect of arena rotation on control performance. Newman–Keuls *post hoc* tests were used where appropriate. Paired *t*-tests were used to assess the effect of injection switch within-subject. No specific corrections for multiple comparisons were performed in this study. These corrections involve a trade-off between Type I and II errors and usually require the comparisons to be independent. Bonferroni correction would accept significance at α = 0.0167, the less conservative Šidák correction at α = 0.0170. Majority of tests reported here are not affected by these adjustments because they yielded much lower *p*. In notable exceptions (see Discussion and MK-801 Impaired Segregation of Discordant Spatial Information), a Tukey HSD test was used as a weak control for Type I error connected with repeated measures. Given that the repeated measures were not independent, we report uncorrected ANOVA statistics and actual values for all *p* > 0.01 to provide maximum relevant information. Five rats were excluded from the analysis. One rat did not receive the shock properly due to a damaged cable, and four rats (each from a different group) were excluded based on Dixon’s *Q*-test for outliers (*Q* = gap/range) at 99% confidence (Rorabacher, 1991; Christian, 2003).

### Immediate-early gene imaging

Thirty rats received two 6 min exploration sessions in either the same environment A/A or in two different environments A/B separated by 20 min (Figure 2D). Environment A was the Carousel arena described above equipped with three identical objects (dark plastic cylinders 8 cm high 2 cm diameter) whereas environment B was a white square open field of equivalent size (72 cm) with three wooden blocks 3 cm × 6 cm × 6 cm located in a different room. Twelve (four per each treatment) more rats served as caged controls (CC). The rats were injected with saline or MK-801 (0.10 and 0.15 mg/kg) 30 min before the exploration sessions. Immediately after the second session, the rats were deeply anesthetized with isoflurane and decapitated. Their brains were quickly removed, flash-frozen in a dry ice-cooled isopentane bath and stored in a −80°C freezer for later analysis. Four millimeter segments containing the dorsal hippocampus from left hemispheres were arranged in blocks maximizing the number of within-block, between-group comparisons and embedded in optimal cutting temperature medium (OCT; Sakura). The blocks were sectioned at 20 μm in a cryostat (Leica CM 1850, Germany), mounted on gelatine-coated superfrost slides (Fisher), and processed for fluorescence *in situ* hybridization as previously described (Vazdarjanova and Guzowski, 2004; Kubik et al., 2012). Briefly, fluorescein-labeled antisense riboprobes for *Homer 1a* 3′UTR and digoxigenin-labeled *Arc* antisense riboprobes were hybridized overnight in a single hybridization step and then sequentially detected with anti-fluorescein-HRP (Jackson labs) and anti-digoxigenin-HRP FAB fragments (Roche). *Homer 1a* probes were visualized with a tyramide-fluorescein signal amplification system (TSA-Fluorescein) and *Arc* probes with TSA-Cy3 (Perkin-Elmer). Slides were incubated with a nuclear counterstain (DAPI, Invitrogen), coverslipped with antifade media (Vectashield, Vector labs), and sealed with nail polish.

### Image data acquisition and analysis

Confocal stacks from CA1 were acquired on an inverted Leica SP5 laser scanning microscope with apochromatic objectives HCX PL APO 20× (n.a. 0.7) imm corr Lbd. BL and HCX PL APO 10× (n.a. 0.40) CS. The blue signal (DAPI) was imaged using 405 nm excitation and a 415–490 bandpass, the green signal (TSA-Fluorescein) with 488 nm excitation and a 510–550 bandpass, and the orange/red signal (TSA-Cy3) with 561 nm excitation and a 610–680 bandpass. The laser power, gain, and offset were always set for the whole slide. The settings were optimized to obtain bright intranuclear foci of ongoing IEG transcription. Six to eight CA1 images with an average of 570 ± 13 cells were analyzed from each animal. The image data were analyzed by a technician blind to the identity of the samples using a custom macro for ImageJ as described before (Kubik et al., 2012). The proportions of *Homer 1a*+ and *Arc*+ neurons were used to map neuronal ensembles active during the first and second test session, respectively. The similarity of these activated ensembles was evaluated using similarity scores = diff(E1E2)/least epoch – *p*(E1E2), where E1 and E2 are proportions of *Homer 1a*+ and *Arc*+ neurons, respectively, *p*(E1E2) is a random overlap = E1*E2, least epoch is the smaller one of E1 and E2, and diff(E1E2) is the difference between the observed proportion of double-labeled neurons (*Arc*&*Homer 1a*)+ and the random overlap (pE1E2) between the two ensembles. A three-way ANOVA with repeated measures on the test sessions (*Arc*+ and *Homer 1a*+), and main factors of treatment (saline, 0.10 and 0.15 mg/kg MK-801), and behavior (A/A, A/B, CC) was used to evaluate the effect of MK-801 on IEG expression as such and specifically after behavioral exploration. A two-way factorial ANOVA on treatment (saline, 0.10 and 0.15 mg/kg MK-801), and behavior (A/A, A/B) as independent variables was used to evaluate the effect of MK-801 on ensemble similarity scores in CA1. Newman–Keuls *post hoc* tests were used when appropriate.

## Results

We first compared the effects of two doses of MK-801 (0.10 and 0.15 mg/kg) on PA on a stable (low coordination demand) or a rotating arena (high coordination demand).

### Room-stable: MK-801 (0.15 mg/kg) spared place avoidance in congruent spatial frames on a stable arena

**Figure 3:**
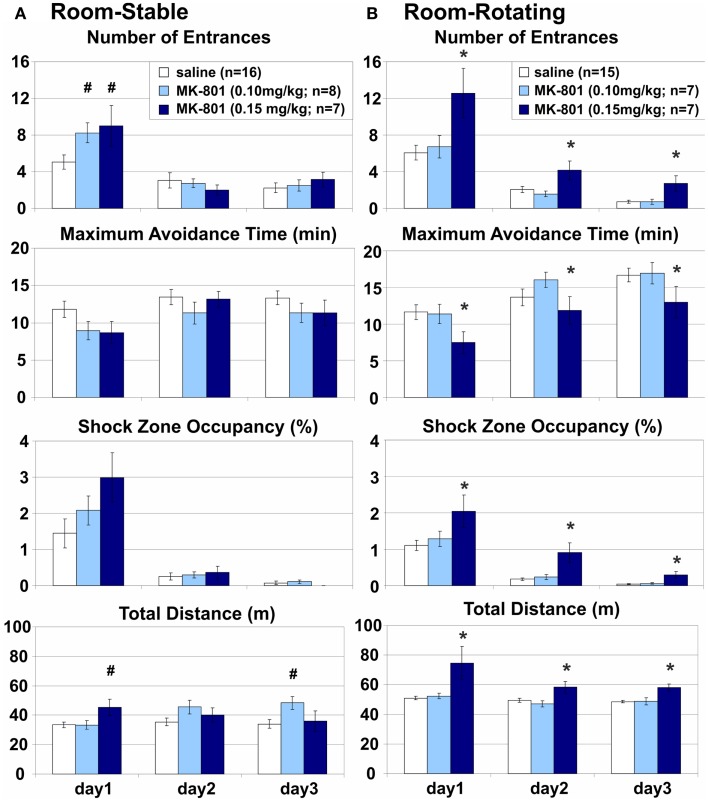
**Room-based place avoidance on a Stable and a Rotating arena**. **(A)** Room-Stable place avoidance on a stationary dry arena with no demand for coordination of information from congruent spatial frames. Only a transient increase in the number of Entrances and a similar trend in the Shock Zone Occupancy were observed on day 1 on the Stable arena after either dose of MK-801. This effect was associated with transient hyperlocomotion after the high, but not the low dose of MK-801. **(B)** Room-Rotating Place Avoidance on a rotating dry arena with a high demand for coordination of information from the dissociated spatial frames. 0.15 mg/kg MK-801 persistently impaired all measures of avoidance and increased locomotion on the Rotating arena compared to the saline controls and 0.10 mg/kg MK-801. All rats improved their performance during training. *Significant effects of treatment; **^#^**significant effect of treatment × session interaction.

Place avoidance on a stable arena was spared when the room and arena frames were not dissociated, all cues could be used to support the avoidance, no misleading information was present, and the coordination demand was low (Figure 3A). No significant main effect of MK-801 treatment was found on any of the parameters on the stationary arena, but significant interactions on *E* (*F*_4, 56_ = 4.64, *p* < 0.005), ED (*F*_4, 56_ = 5.10, *p* < 0.005), and *D* (*F*_4, 56_ = 7.23, *p* < 10^−4^) pointed to a transient impairment on day 1 after the low (*E*: *p* = 0.025; ED: *p* = 0.023) and high dose of MK-801 (*E*: *p* = 0.017). Highly significant effects of days (*E*: *F*_2, 56_ = 49.4, *p* < 10^−6^; MaxT: *F*_2, 56_ = 6.26, *p* < 0.005; pTime: *F*_2, 56_ = 47.1, *p* < 10^−6^; ED: *F*_2, 56_ = 52. 6, *p* < 10^−6^) showed robust learning in all rats during training. However, the learning curve of control animals was relatively flat, especially in the MaxT parameter. To see if this was due to rapid learning on day 1, which was not captured in the between-session comparisons, we compared performance between the two halves of session 1. A two-way repeated measures ANOVA found a dramatic improvement during the session (*E*: *F*_1, 28_ = 55.4, *p* < 10^−6^; MaxT: *F*_1, 28_ = 33.1, *p* < 10^−5^; ED: *F*_1, 28_ = 12.2, *p* < 0.005; pTime: *F*_1, 28_ = 46.2, *p* < 10^−6^).

### Room-rotating: MK-801 (0.15 mg/kg) impaired place avoidance in dissociated spatial frames on a rotating arena

Stationary room-defined PA on a continuously rotating dry arena (Carousel) was impaired when spatial reference frames were dissociated (Figure 3B). Cues from both the room and arena frames were available, but only the room cues predicted the shock while the arena cues were misleading and the coordination demand was high. All PA parameters were markedly impaired (*E*: *F*_2, 26_ = 10.4, *p* < 0.0005; MaxT: *F*_2, 26_ = 5.76, *p* < 0.01; pTime: *F*_2, 26_ = 11.5, *p* < 0.0005; ED: *F*_2, 26_ = 4.26, *p* = 0.025) and locomotion was increased (*D*: *F*_2, 26_ = 10.3, *p* > 0.001) after the high (*E*: *p* < 0.005; MaxT: *p* = 0.011; ED: *p* = 0.016; pTime: *p* < 0.001; *D*: *p* < 0.005), but not the low dose of MK-801 (all *p*s > 0.5), compared to saline controls. An increased number of Entrances per unit Distance suggests that the PA deficit could not be attributed merely to hyperlocomotion (Figure 6; but see Discussion of this parameter in MK-801 Impaired Segregation of Discordant Spatial Information). All rats showed robust between-session learning (*E*: *F*_2, 52_ = 51.8, *p* < 10^−6^; MaxT: *F*_2, 52_ = 11.6, *p* < 10^−4^; TARG: *F*_2, 52_ = 56.8, *p* < 10^−6^; *D*: *F*_2, 52_ = 7.06, *p* < 0.005; ED: *F*_2, 52_ = 57.9, *p* < 10^−6^). Marginally insignificant treatment × session interactions (*E*: *F*_4, 52_ = 2.32, *p* < 0.069; *D*: *F*_4, 52_ = 2.54, *p* < 0.051) reflected a trend toward hyperlocomotion and poorer avoidance after the higher dose of MK-801 specifically in session 1.

Comparison of the performance of control rats between Stable and Rotating arena found no effect of rotation on the avoidance (*E*: *F*_1, 29_ = 0.43, *p* > 0.5; MaxT: *F*_1, 29_ = 1.39, *p* > 0.2; pTime: *F*_1, 29_ = 0.82, *p* > 0.3), but a highly significant effect on locomotion (*D*: *F*_1, 29_ = 37.3, *p* < 10^−5^). Since this effect could confound direct comparisons between different conditions, the effects of MK-801 were always compared to the respective vehicle controls in the same condition. Given the selectivity of the MK-801-induced PA deficit, we focused the following experiments on the effect of the high dose (0.15 mg/kg) on the rotating arena.

### Room-wet: MK-801(0.15 mg/kg) impaired place avoidance on a rotating arena with limited access to irrelevant arena cues

**Figure 4:**
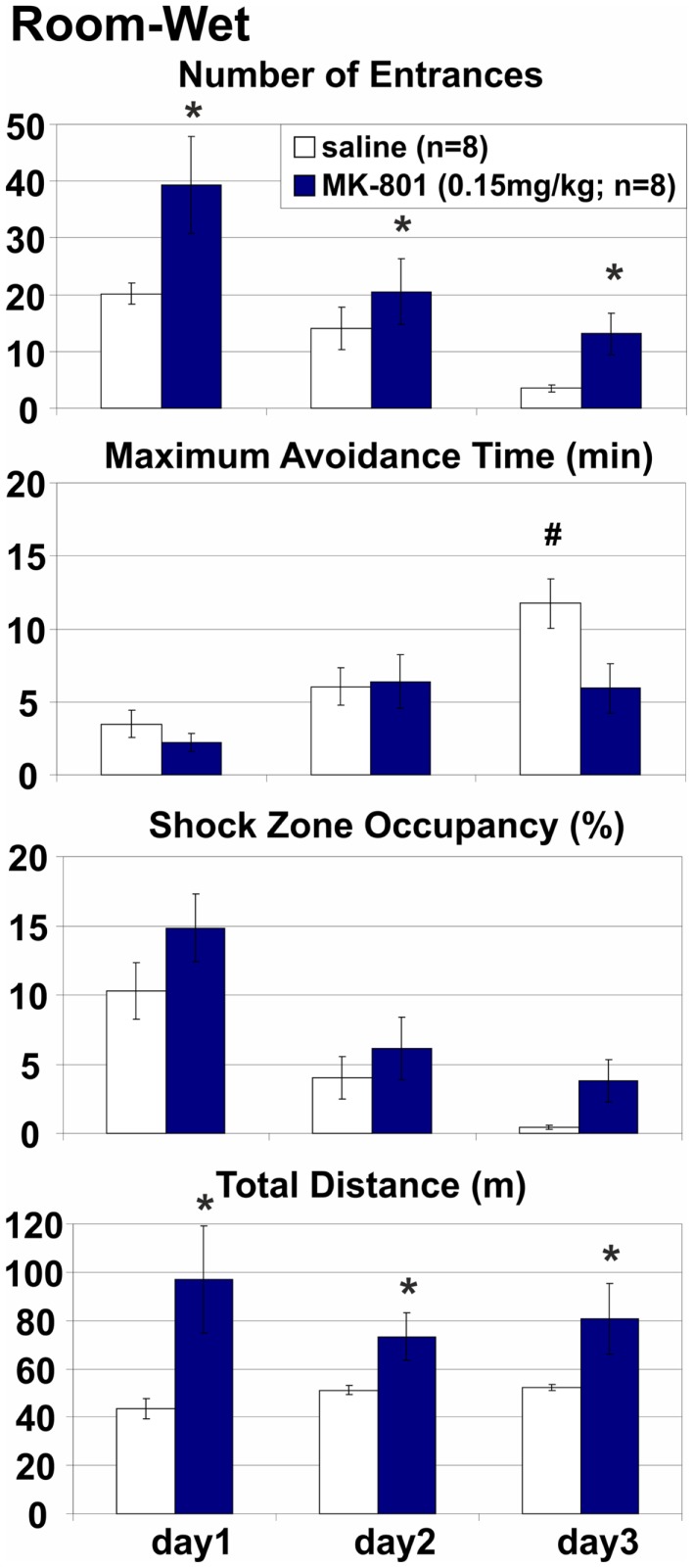
**Room-Wet place avoidance with rotating arena cues obscured by shallow water**. The demand for coordination was reduced but not eliminated because arena-bound visual stimuli, path integration, and idiothetic information remained available. MK-801 (0.15 mg/kg) increased the number of Entrances, but the effect of treatment on other measures of avoidance did not reach significance. All rats improved their performance during training. *Significant effects of treatment; **^#^**significant effect of treatment × session interaction.

Stationary room-defined PA in dissociated spatial frames was impaired when the salience of the rotating arena cues was attenuated by shallow water (Figure 4). Misleading substratal cues on the rotating arena surface were obscured, but arena-bound visual stimuli, path integration, and idiothesis were not removed and could interfere with the avoidance. The coordination demand was reduced, but not eliminated. MK-801 increased the number of Entrances (*E*: *F*_1, 14_ = 7.95, *p* = 0.014) but not the number of Entrances per unit Distance (ED: *F*_1, 14_ = 1.25, *p* > 0.2; Figure 6), indicating that this effect could be related to the observed hyperlocomotion (*D*: *F*_1, 14_ = 11.4, *p* < 0.005). Other parameters were not significantly affected by MK-801 (*p*s > 0.1). Control rats also reached longer Maximum Avoidance Time on day 3. Again, robust learning was observed in all rats during the training (*E*: *F*_2, 28_ = 10.9, *p* < 0.0005; MaxT: *F*_2, 28_ = 16.2, *p* < 10^−4^; pTime: *F*_2, 28_ = 30.9, *p* < 10^−6^; ED: *F*_2, 28_ = 32.0, *p* < 10^−6^). A significant treatment × day interaction on MaxT (*F*_2, 28_ = 4.61, *p* < 0.019) reflected better performance by control rats on day 3 (*p* < 0.05).

### Arena-dark: MK-801 (0.15 mg/kg) spared place avoidance without irrelevant room cues on an arena rotating in darkness

**Figure 5:**
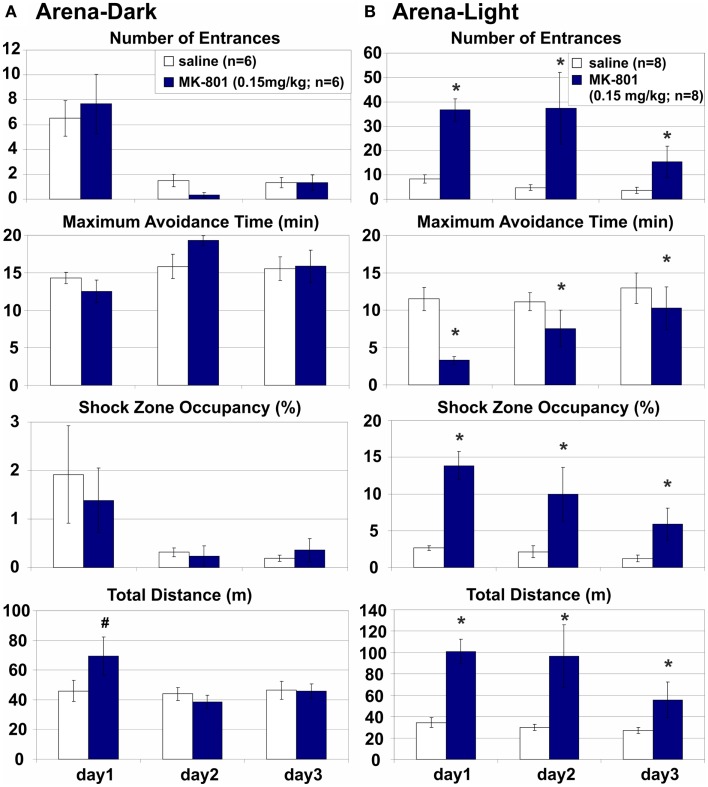
**Arena-based place avoidance on a rotating arena in Darkness and in Light**. **(A)** Arena-Dark place avoidance posed no demand for coordination because the irrelevant room stimuli were hidden by darkness. MK-801 transiently increased locomotion in Darkness on day 1. **(B)** Arena-Light place avoidance with high demand for coordination due to interference from irrelevant room cues In contrast to darkness, MK-801 markedly and persistently impaired all place avoidance measures in Light. All rats improved their performance during training. *Significant effects of treatment; **^#^**significant effect of treatment × session interaction.

While complete removal of arena frame information is difficult (but see Stuchlík et al., 2001), eliminating room-based, predominantly visual cues is very simple. Therefore, we compared Arena-based PA on a dry rotating arena with or without access to irrelevant, misleading visual stimuli in light or darkness, respectively. Avoidance of a rotating arena-defined place was not affected by MK-801 when irrelevant room cues were hidden by darkness and the coordination demand was low (Figure 5A). MK-801 caused a transient hyperlocomotion (*D*: *F*_2, 20_ = 4.37, *p* = 0.027) on day 1 (*p* < 0.01), but did not affect any PA parameter. All rats showed robust learning during training (*E*: *F*_2, 20_ = 17.9, *p* < 10^−4^; MaxT: *F*_2, 20_ = 3.68, *p* = 0.043; pTime: *F*_2, 20_ = 4.85, *p* = 0.019; *D*: *F*_2, 20_ = 8.93, *p* < 0.005; ED: *F*_2, 20_ = 32.7, *p* < 10^−5^).

### Arena-light: MK-801 (0.15 mg/kg) impaired place avoidance in dissociated spatial frames on an arena rotating in the light

**Figure 6:**
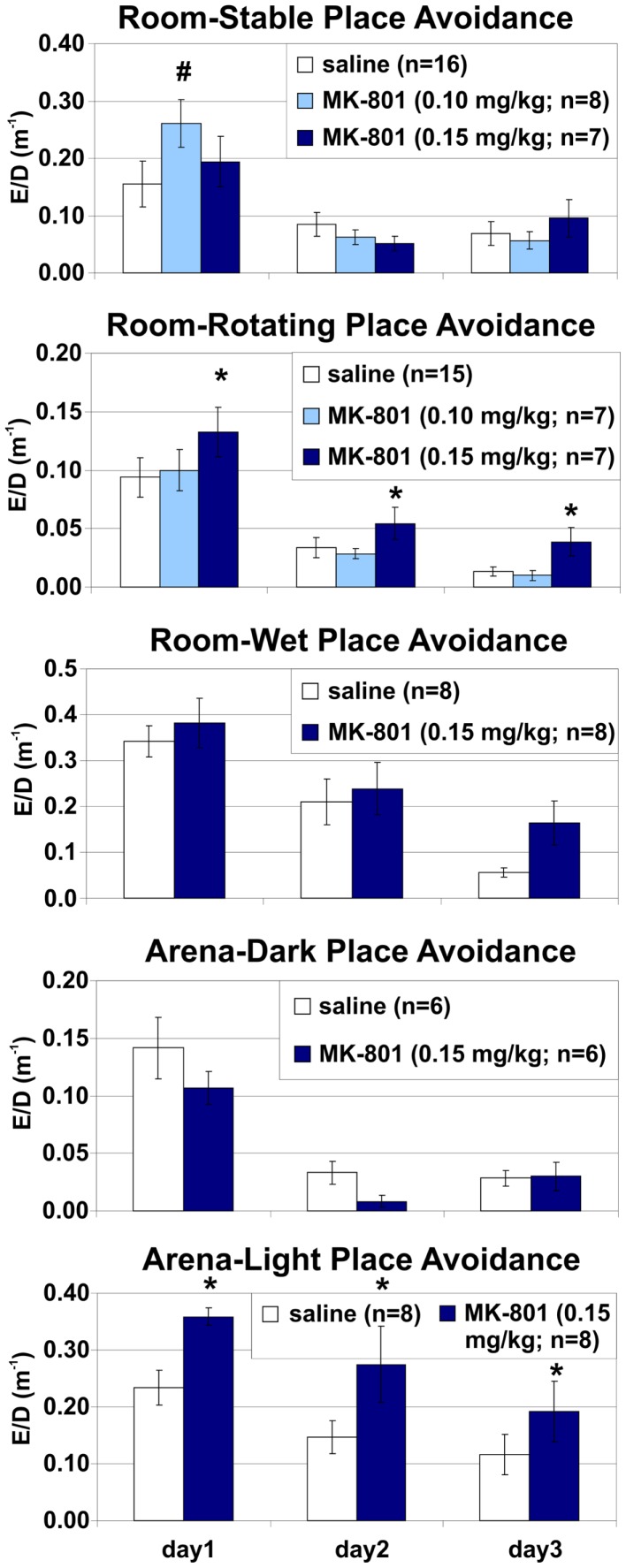
**Place avoidance normalized by locomotion in different versions of the place avoidance task**. MK-801 (0.15 mg/kg) increased the number of entrances per unit distance (ED) in the Room-Rotating and Arena-Light conditions, suggesting that the place avoidance deficit was not due to hyperlocomotion. *Significant effects of treatment; **^#^**significant effect of treatment × session interaction.

Avoidance of an arena-defined place on a rotating arena in the light was severely impaired when irrelevant room cues were available and the coordination demand was high (Figure 5B). MK-801 impaired all PA parameters (*E*: *F*_1, 14_ = 10.6, *p* < 0.01; MaxT: *F*_1, 14_ = 5.28, *p* = 0.037; pTime: *F*_1, 14_ = 12.4, *p* < 0.005; ED: *F*_1, 14_ = 5.34, *p* > 0.05) and increased locomotion (*D*: *F*_1, 14_ = 11.8, *p* < 0.005). A significant effect of MK-801 on the number of Entrances per unit Distance shows hyperlocomotion is an unlikely explanation of the PA deficit (Figure 6; but see Discussion of this parameter in MK-801 Impaired Segregation of Discordant Spatial Information). All rats improved performance during training (*E*: *F*_2, 28_ = 3.45, *p* = 0.046; MaxT: *F*_2, 28_ = 3.88, *p* = 0.033; pTime: *F*_2, 28_ = 5.78, *p* < 0.01; ED: *F*_2, 28_ = 11.0, *p* < 0.0005).

### Extended room-rotating training: Place avoidance is impaired after switching to or from MK-801 (0.15 mg/kg)

**Figure 7:**
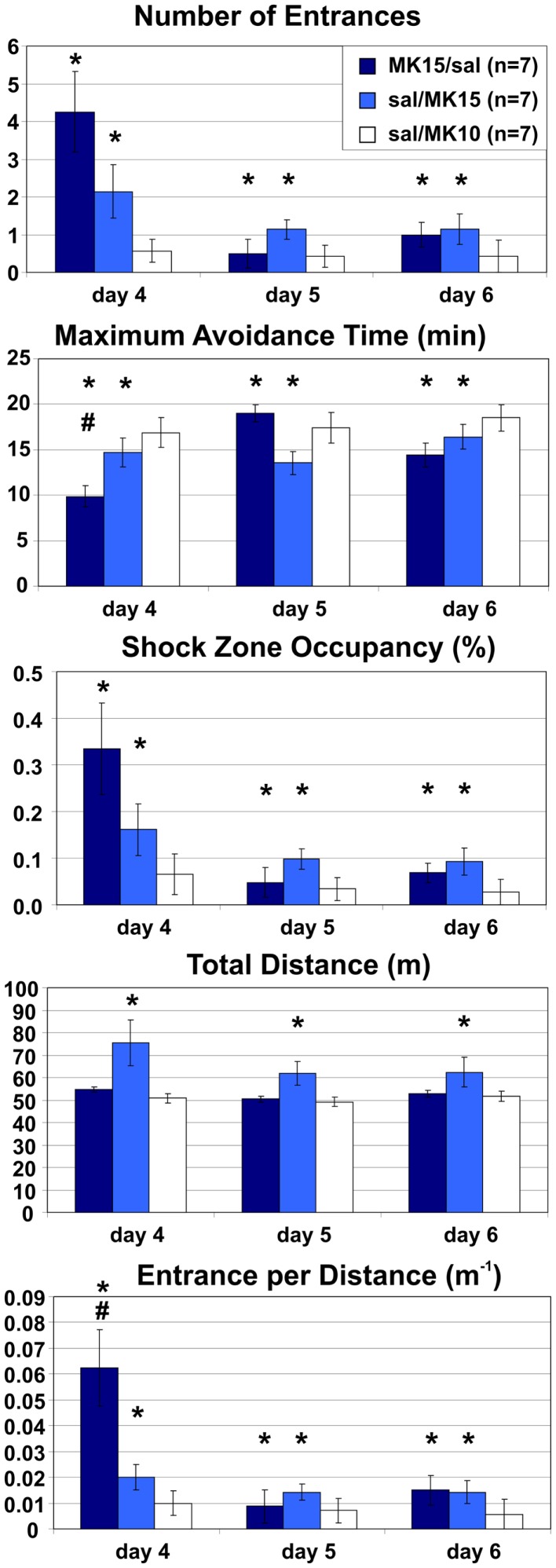
**Room-Rotating place avoidance after reversed injections**. Rats trained with MK-801 (0.15 mg/kg) on days 1–3 received saline on days 4–6 (MK15/sal) and rats initially trained with saline received MK-801 at 0.15 mg/kg (sal/MK15) or 0.10 mg/kg (sal/MK10). Rats in the sal/MK10 group avoided better than the other rats. Locomotion was increased in the sal/MK15 group. Switching from MK-801 to saline caused a marked but transient place avoidance deficit on day 4, which disappeared on the next day (MK/sal). Switching from saline to MK-801 (0.15 mg/kg) caused a less dramatic, but more persistent place avoidance deficit. Significant effects of treatment are marked with asterisk (*) and a significant effect of treatment × session interaction is marked with number sign (**^#^**).

Rats in both MK15/sal and sal/MK15 groups were impaired relative to control animals (sal/MK10). A two-way ANOVA with repeated measures on the three extra days found a significant main effects of groups on all measures (*E*: *F*_2, 18_ = 7.48, *p* < 0.005; MaxT: *F*_2, 18_ = 7.24, *p* < 0.005; pTime: *F*_2, 18_ = 4.05, *p* = 0.035; *D*: *F*_2, 18_ = 9.09, *p* < 0.005; ED: *F*_2, 18_ = 10.0, *p* < 0.005). *Post hoc* tests showed that rats in the sal/MK10 group avoided more than rats in MK15/sal (*E*: *p* < 0.01; MaxT: *p* < 0.01; pTime: *p* = 0.039; ED: *p* = 0.001) and sal/MK15 (*E*: *p* = 0.014; MaxT: *p* < 0.01; pTime: *p* = 0.044) groups. Locomotion was increased in the sal/MK15 group (*D*: *p*s < 0.005). Significant effects of days (*E*: *F*_2, 36_ = 5.71, *p* < 0.01; pTime: *F*_2, 36_ = 5.17, *p* = 0.011; ED: *F*_2, 36_ = 5.15, *p* = 0.011) reflect improved performance on days 5 and 6 and group × day interaction (MaxT: *F*_4, 36_ = 2.86, *p* = 0.037; ED: *F*_4, 36_ = 2.93, *p* = 0.034) show markedly worse performance in the MK15/saline group on day 4. The *post hoc* tests showed that ED was increased compared to other days and groups (all *p*s < 0.005) and MaxT was shorter than on day 2 and 3 of saline/MK15 and all days in the controls (sal/MK10; all *p*s < 0.05). A similar pattern was observed for the marginal interaction on the number of Entrances (*F*_4, 36_ = 2.29, *p* = 0.078), suggesting that the group effect for MK15/sal rats may be driven by impaired performance after the switch on day 4. In contrast, rats initially trained with saline appeared to display a less dramatic, but more sustained deficit after switching to 0.15 mg/kg MK-801 (sal/MK15; Figure 7).

**Figure 8:**
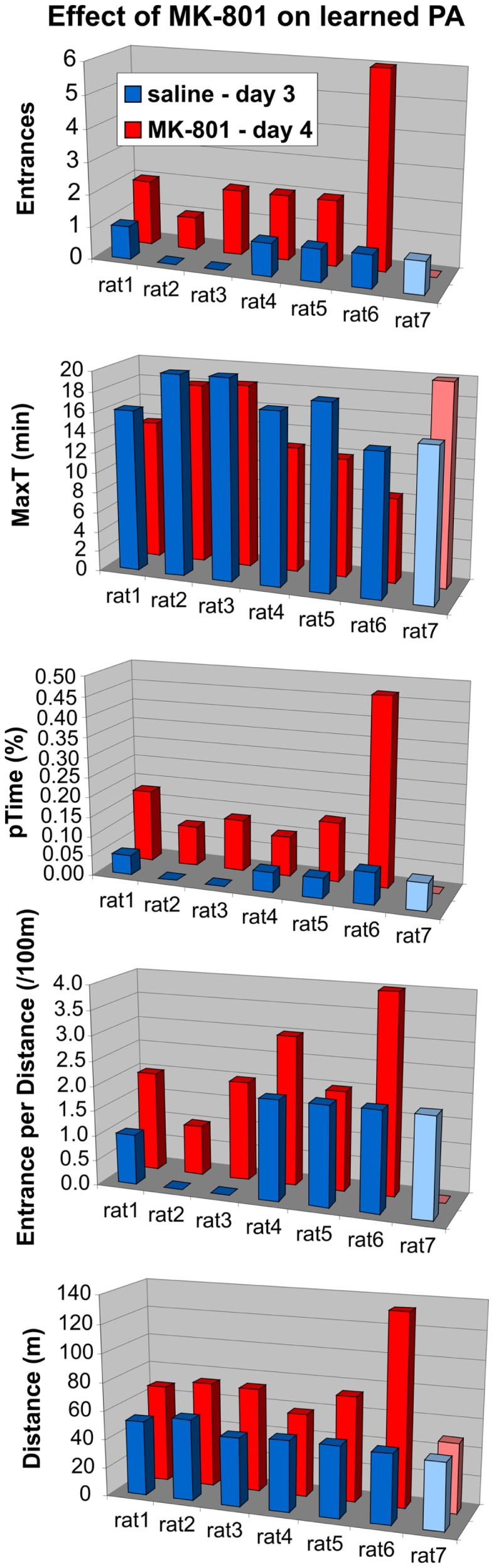
**Effect of MK-801 (0.15 mg/kg) on well-trained avoidance**. MK-801 impaired avoidance and increased locomotion in all animals (sal/MK15) except rat 7. Rat 6 deteriorated approximately three times as much as other rats (except MaxT).

**Figure 9:**
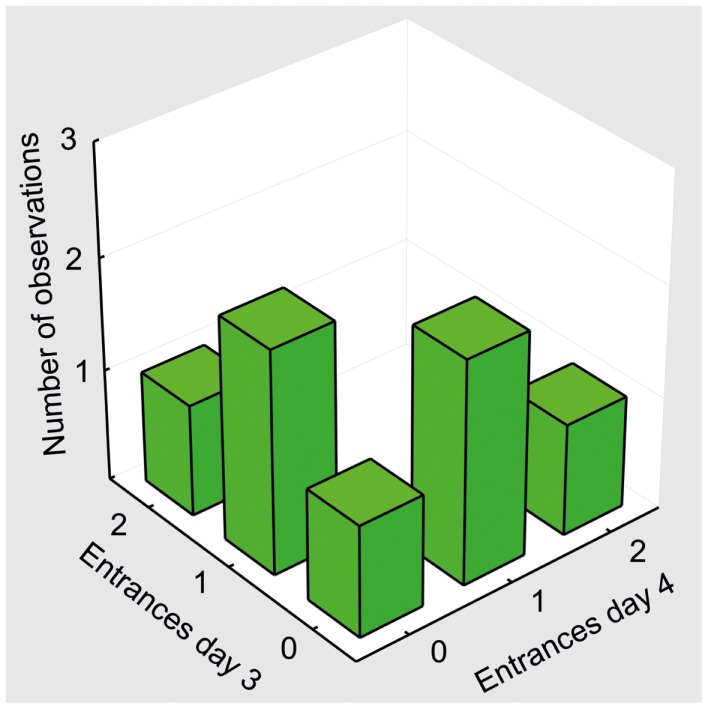
**Behavioral pattern in control animals (sal/MK10)**. A bivariate histogram for the number of Entrances on days 3 and 4. No rat entered the shock zone in both sessions. One entrance in one of the sessions was the most typical performance in the control group.

To see if this is indeed the case, we compared their performance to controls selectively on days 5 and 6. The ANOVA (sal/MK15 vs. sal/MK10) found a significant group effect on pTime (*F*_1, 12_ = 7.44, *p* = 0.018) and marginal effects on Entrances (*F*_1, 12_ = 4.23, *p* = 0.062) and MaxT (*F*_1, 12_ = 4.72, *p* = 0.051). No such effects were found for the MK15/sal rats (all *p*s > 0.26) suggesting that in contrast to the MK15/sal group, the effect of group on the 3-day performance was not driven solely by impairment on day 4 in the sal/MK15 group. To examine the immediate effect of MK-801 on well-learned PA within-subject, we compared performance on days 3 and 4 with paired *t*-tests. The analysis yielded only a marginally insignificant difference for the sal/MK15 group (E: *p* = 0.082; pTime: *p* = 0.064) and no difference in the MK15/sal group (all *p* > 0.25) and controls (sal/MK10; all *p* > 0.59). This result was puzzling given the significant difference between the sal/MK15 and the controls (sal/MK10) revealed by the ANOVA on days 4–6 of the extended training. We examined individual performance (Figure 8) to see if excessive variance could explain the lack of effect between days 3 and 4. Avoidance deteriorated and locomotion increased after switching to 0.15 mg/kg MK-801 in all rats except one (#7), which improved avoidance with no change in locomotion. This rat made one error on day 3 and none on day 4 resembling behavior of control rats (sal-to-MK10), which only made error(s) in one session, but never in both (Figure 9). Without this outlier (with respect to the general effect of 0.15 mg/kg MK-801), the *t*-tests were significant for all avoidance parameters in the sal/MK15 group (E: *p* = 0.038; MaxT: *p* < 0.01; pTime: *p* = 0.029; E/D: *p* = 0.013; D: *p* = 0.052). In addition, disproportional impairment in rat #6 represents another major source of variance as bringing the values closer to the group means restored significances for E and pTime. Combined, these two manipulations brought the *t*-tests on E and pTime to *p* < 0.005.

### MK-801 reduced IEG expression in the hippocampus

**Figure 10:**
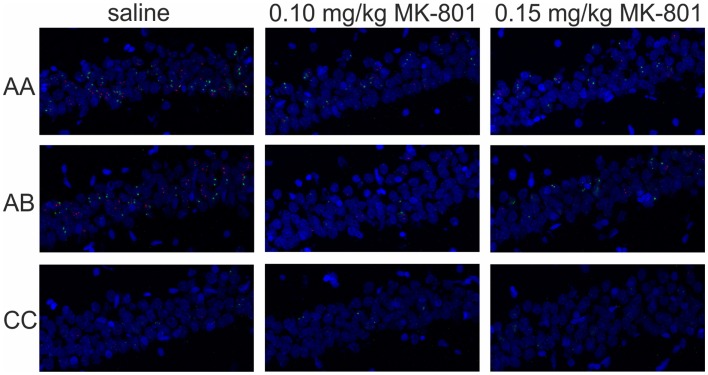
**Immediate-early gene expression in CA1**. Representative images showing expression of *Homer 1a* (green) and *Arc/Arg3.1* (red) triggered in the CA1 region of the hippocampus by exploration of the same (AA) or two different environments (AB) after an injection of saline or MK-801 (0.10 and 0.15 mg/kg).

**Figure 11:**
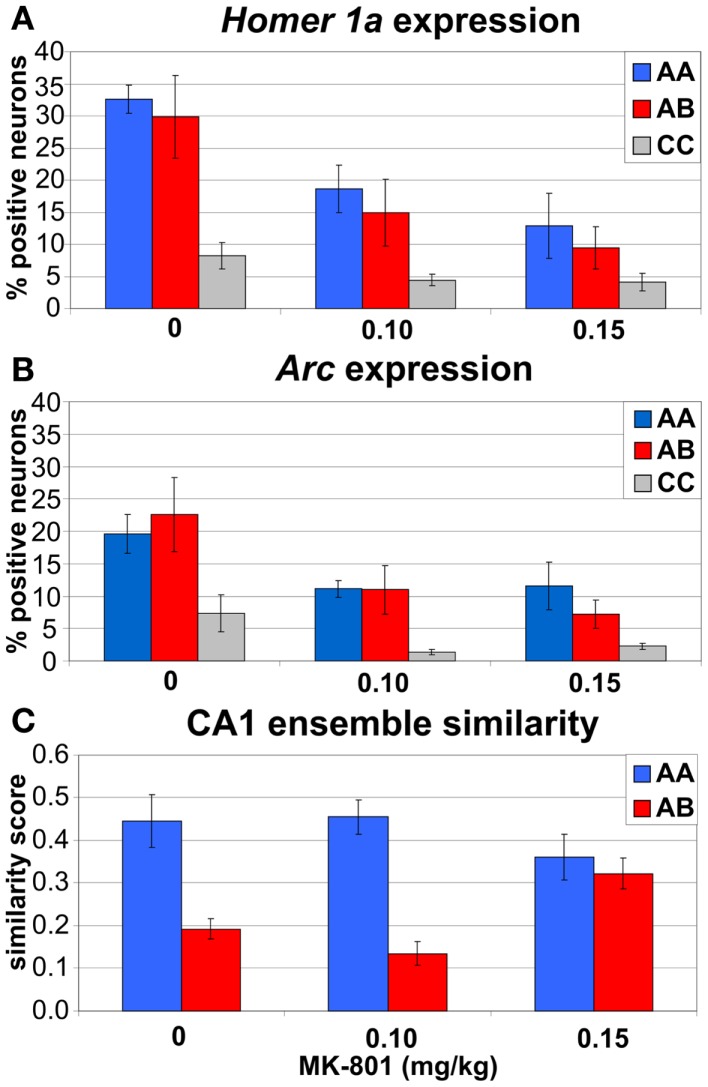
**Immediate-early gene imaging data summary**. **(A,B)** both doses of MK-801 reduced expression of IEGs *Homer1a* **(A)** and *Arc* **(B)** in CA1 neurons. **(C)** Higher dose of MK-801 (0.15 mg/kg) increased similarity between CA1 ensembles activated in different environments **(A,B)** and eliminated the contextual specificity of the IEG expression observed after saline and 0.10 mg/kg MK-801.

The effects of MK-801 on IEG expression in CA1 are illustrated in Figure 10 and the IEG expression data analysis is summarized in Figure 11.

The three-way ANOVA found significant main effects of treatment (*F*_2, 33_ = 10.7, *p* < 0.0005), behavior (*F*_2, 33_ = 12.1, *p* < 0.0005), and session (*F*_1, 33_ = 21.7, *p* < 10^−4^), but no interaction (Figures 11A,B). *Post hoc* tests showed that both doses of MK-801 substantially reduced the IEG expression (*p*s < 0.001), that behavioral experience (A/A, A/B) dramatically increased the IEG expression compared to CC (*p*s < 0.0005), and that the IEG expression in session 2 was lower than in session 1 (*p*s < 0.0005). This last effect appeared to be driven largely by saline and 0.10 mg/kg MK-801 treatments in behaving animals (A/A and A/B) as opposed to 0.15 mg/kg MK-801 treatment and CC, but none of the interactions reached significance. To see if it could be due to large heterogeneity of the sample, we analyzed the expression separately in the behaving and CC animals.

### MK-801 (0.15 mg/kg) prevented habituation of the IEG expression response

To examine the effect of MK-801 specifically on the behaviorally induced IEG expression, we focused the analysis on animals, which explored environments A/A and A/B during the test sessions. A three-way ANOVA found significant main effects of treatment (*F*_2, 24_ = 9.48, *p* < 0.001) and session (*F*_1, 24_ = 25.4, *p* < 10^−4^), and a significant treatment × session interaction (*F*_2, 24_ = 4.35, *p* = 0.024), but no effect of behavior (*F*_1, 24_ = 0.36, *p* > 0.5). *Post hoc* tests showed that the IEG expression decreased after both doses of MK-801 (*p*s < 0.005) and that it was significantly lower in the second session than in the first in rats treated with saline (*p* < 0.0005) and 0.10 mg/kg (*p* = 0.025), but not 0.15 mg/kg MK-801 (*p* > 0.6). A separate two-way ANOVA on the IEG expression in CC animals found a significant effect of treatment (*F*_2, 9_ = 21.3, *p* < 0.0005), but no effect of session, and no interaction. *Post hoc* tests showed that both doses of MK-801 (*p*s < 0.001) reduced IEG expression in CC.

### MK-801 (0.15 mg/kg) increased similarity between unrelated CA1 ensembles

To examine the effect of MK-801 treatment specifically on differences in CA1 ensemble similarity between A/A and A/B conditions, we analyzed similarity scores between CA1 ensembles expressing IEGs during sessions 1 and 2. A two-way ANOVA found a significant main effect of behavior (*F*_1, 24_ = 34.1, *p* < 10^−4^) and a significant treatment × behavior interaction (*F*_2, 24_ = 5.96, *p* < 0.01), but no significant main effect of treatment (*F*_2, 24_ = 0.59, *p* > 0.5). *Post hoc* tests showed that the similarity was lower in A/B than in A/A condition after saline (*p* < 0.005) and 0.10 mg/kg MK-801 (*p* < 0.0005). In contrast, after 0.15 mg/kg MK-801, similarity in A/B was equivalent to A/A (*p* > 0.5) and significantly higher than in A/B condition after saline (*p* = 0.013) or 0.10 mg/kg MK-801 (*p* = 0.042; Figure 11C).

## Discussion

### MK-801 impaired segregation of discordant spatial information

MK-801 (0.15 mg/kg) impaired PA when irrelevant information was present (Room-Rotating and Arena-Light), but not when it was absent (Room-Stable or Arena-Dark). These PA versions differed in their demand for segregation of relevant and irrelevant spatial information, but not in mnemonic or motivational demands, demonstrating that general learning impairment, selective impairment of either allothetic or idiothetic (egocentric) navigation, or MK-801-induced alterations in motivation or shock sensitivity cannot explain the PA deficit. Partial PA deficit was also observed when cues on the arena surface (such as scent marks, urine, or droppings) were obscured by shallow water. Note that misleading, Arena-bound cues, related to idiothesis and path integration were not affected by the water and could interfere with the Room-based avoidance. Poorer avoidance on the wet arena is likely due to the water reduced impedance between a rat and the grounded arena and alleviated perception of shock in the constant-current circuit. Indeed, despite the shock intensity was often set to the maximum of 0.7 mA, the control rats made many more errors and received many more shocks (~60) than in any dry arena version (10–15) on PA day 1. By day 3, however, the controls’ avoidance matched other versions (entrances: 3.5 vs. 0.73–3.625; shocks: 4.125 vs. 0.73–8.0). Locomotion was slightly but consistently lower on the wet arena (~30 compared to 40 m on dry arena) during foraging before the first injection (version: *F*_1, 36_ = 21.2, *p* < 10^−4^). MK-801 increased locomotion on foraging day 4 (treatment: *F*_1, 34_ = 11.7, *p* < 0.005), particularly on the wet arena (~80 vs. 45 m; interaction *F*_1, 34_ = 4.9, *p* < 0.05). During avoidance, shallow water did not affect the locomotion of control rats, but accentuated MK-801-induced hyperlocomotion. The controls covered, on average, 43–52 m on the wet, compared to ~50 m on the dry rotating arena. MK-801 (0.15 mg/kg) increased that to 80–97 m on the wet and 58–74 m on the dry rotating arena (Room-Rotating; Figures 3 and 4). In agreement with previous reports (Stuchlík et al., 2004; Valeš et al., 2006), the low dose of MK-801 (0.10 mg/kg) had a little effect on the PA. Rotation of the arena itself did not affect the PA in control rats, but it substantially facilitated locomotion, presumably by continuously bringing rats toward the Shock zone. This pro-locomotory drive was absent on the stable arena. Hyperlocomotion invariably accompanied the PA deficit, but it was absent when PA was unaffected. Different locomotory demands could explain differences in locomotion and avoidance between the Stable and Rotating arena, but not between Light and Darkness. Furthermore, MK-801 impaired the number of Entrances per unit Distance, suggesting that hyperlocomotion does not suffice to explain the PA deficit (Figure 6). However, it should be noted that this effect was not significant after Bonferroni (α = 0.0167) or Šidák correction (α = 0.0170) for multiple comparisons in either the Room-Rotating (*F*_2, 26_ = 4.26, *p* = 0.025) or the Arena-Light version (*F*_1, 14_ = 5.34, *p* = 0.037). On the other hand, both comparisons proved significant using Tukey’s HSD test (*p* = 0.029 and *p* = 0.037, respectively) as a weak control of Type I error connected with repeated measures. Together, the dependence of the MK-801-induced hyperlocomotion on the PA deficit and the effect of MK-801 on ED suggest that, rather than causing the PA deficit, the accompanying hyperlocomotion results from an inability to efficiently organize spatial behavior in dissociated reference frames.

Well-learned PA was impaired when MK-801 (0.15 mg/kg) was injected instead of saline during the extended training. The *t*-test analysis fell short of significance, but this shortcoming was partly due to too much impairment rather than too little and partly due to a single animal, which displayed neither deteriorated avoidance nor increased locomotion after switching to 0.15 mg/kg MK-801 and resembled behavioral pattern observed in controls. Given these were the main sources of variance responsible for the lack of significance in the primary *t*-test analysis, we conclude that the MK-801-induced PA deficit was not selective to learning. In summary, our behavioral data provide compelling evidence that 0.15 mg/kg MK-801 disrupted segregation of discordant spatial information into coherent representations on the Carousel.

That is not to imply that cognitive coordination is the only function affected by MK-801. Low doses of up to 0.10 mg/kg MK-801 impaired cognitive performance of Wistar rats without causing gross sensorimotor or motivational disturbances in a variety of behavioral tasks (van der Staay et al., 2011). The lack of effect of the low dose in this study may be due to higher sensitivity of Wistar rats to the effects of MK-801 (Valeš et al., 2006) or due to lower sensitivity of the PA task compared to spatial water maze (WM) (Stuchlík et al., 2004). MK-801 also induced social withdrawal in a model of negative symptoms of schizophrenia (Rung et al., 2005) and impaired visuospatial working memory (Zemanova et al., 2013) and reversal learning on the Carousel (Lobellova et al., 2013). Importantly, MK-801 also slightly reduced sensitivity to electric shock (van der Staay et al., 2011); therefore, we adjusted the shock intensity individually for each rat based on behavioral markers (escape response) to enhance comparability between the groups.

### Cognitive coordination in the hippocampus

The notion that the PA on the Carousel depends on cognitive coordination of room and arena cues is based on several observations. First, PA acquired on a stationary arena can later be expressed and extinguished separately in the arena frame in darkness, and in the room frame on a rotating arena in light (Bureš et al., 1997; Fenton et al., 1998). Second, hippocampal place cells represent locations in both room and arena frames dissociated by rotation (Zinyuk et al., 2000; Fenton et al., 2010; Kelemen and Fenton, 2010). Third, hippocampal ensemble activity rapidly switches between multiple representations of the same space on a rotating arena (Kelemen and Fenton, 2013) and during a “teleportation” task where two sets of cues are alternated within a single physical environment (Ježek et al., 2011). Fourth, injection of TTX into one hippocampus prevented the PA when room and arena frames were dissociated by rotation and misleading cues were present, but not when they were absent (Wesierska et al., 2005). When cues on the arena surface were obscured by shallow water, rats could perform the previously acquired PA, but new PA learning was not possible after the TTX injection (Kubik and Fenton, 2005). In contrast, retrieval of an existing memory trace was impaired but new spatial learning was spared in the WM after partial hippocampal inactivation (Fenton and Bureš, 1993; Moser and Moser, 1998) suggesting that WM and PA engage distinct hippocampal functions, spatial representation, and segregation, respectively (Kubik and Fenton, 2005). Specifically, whereas locating a small platform (1/330 area) in a large WM requires precise spatial representation, a relatively coarse spatial representation may be sufficient to support avoidance of a large (1/6 area) place on the rotating arena, provided that dissociated room- and arena-frame information can be segregated into coherent subsets. These findings demonstrated that multiple representations of the same space are coordinated within hippocampal neural activity.

### IEG imaging of hippocampal ensemble similarity

In agreement with previous reports (Guzowski et al., 1999; Vazdarjanova and Guzowski, 2004), more similar CA1 ensembles expressed the IEGs in the same environment (A/A) than in different environments (A/B) in rats injected with saline or the low dose of MK-801 (0.10 mg/kg). This contextual specificity was eliminated after the high dose of MK-801 (0.15 mg/kg), which also impaired cognitive coordination on the Carousel. Specifically, the ensemble similarity was increased in A/B, but not in A/A. Decreased IEG expression after either dose of MK-801 could be due to inhibition of NMDA receptors on pyramidal neurons and their role in inducing IEG expression and synaptic plasticity (Link et al., 1995; Steward and Worley, 2001; Czerniawski et al., 2011). On the other hand, the loss of contextual specificity was only observed after the high dose and it may be more closely related to the psychotomimetic effect of MK-801, presumably via its effect on NMDARs on inhibitory interneurons (Grunze et al., 1996; Li et al., 2002). *Arc* expression can be induced in hippocampal CA3 neurons rapidly enough to support one-trial memory formation. The expression is abolished by inactivation of medial septum known to disrupt hippocampal plasticity and learning (Miyashita et al., 2009). Interfering with *Arc* expression impairs maintenance of synaptic plasticity and memory consolidation (Guzowski et al., 2000; Plath et al., 2006), suggesting that *Arc* expression marks plasticity-inducing neural activity. Although the effects of systemic MK-801 are definitely not limited to the hippocampus, our IEG imaging data match the prediction of the Hypersynchrony theory and suggest that MK-801 induced coactivity and “co-plasticity” between hippocampal ensembles, which were previously not related. This mechanism could lead to hyperassociation observed in psychosis (Miller, 1989).

### Hypersynchrony and cognitive disorganization

The Hypersynchrony theory posits that behavioral cognitive coordination depends on selective activation of relevant neural representations and suppression of the irrelevant ones (neural coordination). Increasing coactivity between neurons that would normally not fire together impairs neural coordination and causes psychotic disorganization (Fenton, 2009). Several observations support this hypothesis. First, TTX injection into one hippocampus produced transient disinhibition in the uninjected hippocampus and increased coactivity between pairs of CA1 neurons, which did not fire together before the injection, whereas coactivity between previously coactive neurons was not affected (Olypher et al., 2006). This effect could be due to disrupted feed-forward inhibition mediated by the commissural projection (Buzsáki and Eidelberg, 1981). Second, impaired inhibition could also be responsible for the effects of NMDAr antagonists. Fast-spiking inhibitory interneurons display disproportionately higher sensitivity to NMDAr antagonists (Grunze et al., 1996; Li et al., 2002), which cause a disinhibition of pyramidal neurons (Breier et al., 1997; Vollenweider et al., 1997; Jackson et al., 2004; Lisman et al., 2008), increase interference between memories (Chrobak et al., 2008), and decrease cognitive flexibility (Nikiforuk and Popik, 2012). Third, cognitive coordination on the Carousel was disrupted and coactivity between hippocampal pyramidal neurons was increased after 5 mg/kg, but not 3 mg/kg, phencyclidine (PCP) another non-competitive NMDAr antagonist (Fenton et al., 2006). Fourth, PA on the Carousel and accompanying interhippocampal synchrony are also disrupted in a neurodevelopmental model of schizophrenia by neonatal lesions of the ventral hippocampus (Lee et al., 2012).

### Hyperassociation in schizophrenia

Alterations in the hippocampus (Weinberger, 1999; Harrison, 2004), and hippocampus-dependent memory deficits are typical for schizophrenia (Weiss et al., 2003; Boyer et al., 2007; Ragland et al., 2007; Ranganath et al., 2008; Tamminga et al., 2010). Aberrant inhibitory neurotransmission and loss of parvalbumin (PV)-positive interneurons is found in the hippocampus and the dorsolateral prefrontal cortex (Lewis et al., 2005; Benes et al., 2007; Adell et al., 2012). Reduced PV expression was observed in an animal model by repeated (Braun et al., 2007) or acute administration of MK-801 (Romón et al., 2011). Schizophrenia-like phenotype emerged in post-adolescent mice with a genetic ablation of the NMDA receptor function predominantly in PV+ cortical and hippocampal GABAergic interneurons during early postnatal development (Belforte et al., 2010). Reduced neurotransmission along the mossy fiber pathway from the dentate gyrus (DG) to areas CA3 and CA4 of the hippocampus emerge as a key pathophysiology in schizophrenia (Kerwin et al., 1990; Nowakowski et al., 2002; Kolomeets et al., 2005, 2007; Yamasaki et al., 2008; Kobayashi, 2009; Tamminga et al., 2012). Given the role of the DG in pattern separation (PS; Gilbert et al., 2001; Leutgeb et al., 2007; McHugh et al., 2007; Bakker et al., 2008), reduced DG output could disrupt the dynamic balance between PS and pattern completion (PC) in CA3 (Guzowski et al., 2004) and facilitate cross-linking between hippocampal representations of unrelated events. A hyperassociative hippocampal memory system can give rise to inappropriate associations, distorted perception, irrational expectations, and even hallucinations (Behrendt, 2010). In agreement with this perspective, patients with schizophrenia display excessive associations (Miller, 1989; Wentura et al., 2008; Manschreck et al., 2012), increased interference (Westerhausen et al., 2011), reduced cognitive flexibility (Elliott et al., 1998), and autonoetic awareness (Danion et al., 1999), lack of semantic specificity (Tüscher et al., 2005), difficulties to represent expected reward value (Gold et al., 2012), and deficits in emotional discrimination (Schneider et al., 2006).

## Conclusion

Cognitive dysfunction precedes the manifestation of psychotic symptoms from early childhood (Reichenberg et al., 2010; Sørensen et al., 2010) and cognitive performance is the strongest predictor of the functional outcome in schizophrenia (Green, 1996; Green and Nuechterlein, 1999; Green et al., 2004; Bowie et al., 2006; Keefe et al., 2006; Rosenheck et al., 2006). However, cognitive benefits of available antipsychotics remain rather limited (Harvey and Keefe, 2001; Mishara and Goldberg, 2004; Hill et al., 2010). Cognitive disorganization has been proposed as a core cognitive deficit in schizophrenia (Phillips and Silverstein, 2003). The present data demonstrate that systemic MK-801 impairs cognitive coordination on the Carousel and increases similarity between unrelated hippocampal ensemble representations. In addition, it suggests that increased coactivity in previously unrelated neurons may translate into synaptic plasticity and result in hyperassociative psychotic memories. These findings support the Hypersynchrony theory, validate the PA on the Carousel as test of psychosis-related cognitive disorganization, and demonstrate that the concept of cognitive coordination in schizophrenia can be straightforwardly targeted in an animal model.

## Conflict of Interest Statement

The authors declare that the research was conducted in the absence of any commercial or financial relationships that could be construed as a potential conflict of interest.

## Abbreviations

Arc/Arg 3.1, activity-regulated cytoskeleton-associated protein/RNA; CC, caged controls; D, total distance actively traveled during the session; DAPI, 4′,6-diamidino-2-phenylindole; DG, dentate gyrus; E, the number of entrances; ED, the number of entrances per unit distance; FAB, fragment antigen-binding; FISH, fluorescence *in situ* hybridization; GABA, gamma-aminobutyric acid; HRP, horseradish peroxidase; IEG, immediate-early gene; MaxT, maximum avoidance time (duration); NMDA(r), *N*-methyl d-aspartate (receptor); OCT, optimal cutting temperature freezing medium; PA, place avoidance; PV, parvalbumin; pTime, shock zone occupancy; TSA, tyramide signal amplification; TTX, tetrodotoxin; UTR, untranslated region.

## References

[B1] Adell A. Jiménez-Sánchez L. López-Gil X. Romón T. (2012). Is the acute NMDA receptor hypofunction a valid model of schizophrenia? Schizophr. Bull. 38, 9–14 10.1093/schbul/sbr133 21965469 PMC3245580

[B2] Bakker A. Kirwan C. B. Miller M. Stark C. E. L. (2008). Pattern separation in the human hippocampal CA3and dentate gyrus. Science 319, 1640–1642 10.1126/science.1152882 18356518 PMC2829853

[B3] Barch D. M. (2005). The cognitive neuroscience of schizophrenia. Annu. Rev. Clin. Psychol. 1, 321–353 10.1146/annurev.clinpsy.1.102803.143959 17716091

[B4] Behrendt R. P. (2010). Contribution of hippocampal region CA3 to consciousness and schizophrenic hallucinations. Neurosci. Biobehav. Rev. 34, 1121–1136 10.1016/j.neubiorev.2009.12.009 20034516

[B5] Belforte J. E. Zsiros V. Sklar E. R. Jiang Z. Yu G. Li Y. (2010). Postnatal NMDA receptor ablation in corticolimbic interneurons confers schizophrenia-like phenotypes. Nat. Neurosci. 13, 76–83 10.1038/nn.2447 19915563 PMC2797836

[B6] Benes F. M. Lim B. Matzilevich D. Walsh J. P. Subburaju S. Minns M. (2007). Regulation of the GABA cell phenotype in hippocampus of schizophrenics and bipolars hippocampal GABA alterations in schizophrenia. Proc. Natl. Acad. Sci. U.S.A. 104, 10164–10169 10.1073/pnas.0703806104 17553960 PMC1888575

[B7] Bowie C. R. Reichenberg A. Patterson T. L. Heaton R. K. Harvey P. D. (2006). Determinants of real-world functional performance in schizophrenia subjects: correlations with cognition, functional capacity, and symptoms. Am. J. Psychiatry 163, 418–425 10.1176/appi.ajp.163.3.418 16513862

[B8] Boyer P. Phillips J. L. Rousseau F. L. Ilitvitsky S. (2007). Hippocampal abnormalities and memory deficits: new evidence of a strong pathophysiological link in schizophrenia. Brain Res. Rev. 54, 92–112 10.1016/j.brainresrev.2006.12.008 17306884

[B9] Braun I. Genius J. Grunze H. Bender A. Möller H. J. Rujescu D. (2007). Alterations of hippocampal and prefrontal GABAergic interneurons in an animal model of psychosis induced by NMDA receptor antagonism. Schizophr. Res. 97, 254–263 10.1016/j.schres.2007.05.005 17601703

[B10] Breier A. Malhotra A. K. Pinals D. A. Weisenfeld N. I. Pickar D. (1997). Association of ketamine-induced psychosis with focal activation of the prefrontal cortex in healthy volunteers. Am. J. Psychiatry 154, 805–811 9167508 10.1176/ajp.154.6.805

[B11] Bubeníková-Valešová V. Horacek J. Vrajova M. Höschl C. (2008a). Models of schizophrenia in humans and animals based on inhibition of NMDA receptors. Neurosci. Biobehav. Rev. 32, 1014–1023 10.1016/j.neubiorev.2008.03.012 18471877

[B12] Bubeníková-Valešová V. Stuchlík A. Svoboda J. Bureš J. Valeš K. (2008b). Risperidone and ritanserin but not haloperidol block effect of dizocilpine on the active allothetic place avoidance task. Proc. Natl. Acad. Sci. U.S.A. 105, 1061–1066 10.1073/pnas.0711273105 18195350 PMC2242716

[B13] Bureš J. Fenton A. A. Kaminsky Y. Rossier J. Sacchetti B. Zinyuk L. (1997). Dissociation of exteroceptive and idiothetic orientation cues: effect on hippocampal place cells and place navigation. Philos. Trans. R. Soc. Lond. B Biol. Sci. 352, 1515–1524 10.1098/rstb.1997.0138 9368940 PMC1692058

[B14] Buzsáki G. Eidelberg E. (1981). Commissural projection to the dentate gyrus of the rat: evidence for feed-forward inhibition. Brain Res. 230, 346–350 10.1016/0006-8993(81)90413-3 7317783

[B15] Christian G. D. (2003). Analytical Chemistry, 6th Edn New York: Wiley

[B16] Chrobak J. J. Hinman J. R. Sabolek H. R. (2008). Revealing past memories: proactive interference and ketamine-induced memory deficits. J. Neurosci. 28, 4512–4520 10.1523/JNEUROSCI.0742-07.2008 18434529 PMC6670951

[B17] Cimadevilla J. M. Wesierska M. Fenton A. A. Bureš J. (2001). Inactivating one hippocampus impairs avoidance of a stable room-defined place during dissociation of arena cues from room cues by rotation of the arena. Proc. Natl. Acad. Sci. U.S.A. 98, 3531–3536 10.1073/pnas.051628398 11248112 PMC30687

[B18] Czerniawski J. Ree F. Chia C. Ramamoorthi K. Kumata Y. Otto T. A. (2011). The importance of having Arc: expression of the immediate-early gene Arc is required for hippocampus-dependent fear conditioning and blocked by NMDA receptor antagonism. J. Neurosci. 31, 11200–11207 10.1523/JNEUROSCI.2211-11.2011 21813681 PMC6623359

[B19] Danion J. M. Rizzo L. Bruant A. (1999). Functional mechanisms underlying impaired recognition memory and conscious awareness in patients with schizophrenia. Arch. Gen. Psychiatry 56, 639–644 10.1001/archpsyc.56.7.639 10401510

[B20] Deutsch S. I. Rosse R. B. Billingslea E. N. Bellack A. S. Mastropaolo J. (2002). Topiramate antagonizes MK-801 in an animal model of schizophrenia. Eur. J. Pharmacol. 449, 121–125 10.1016/S0014-2999(02)02041-1 12163115

[B21] Ellenbroek B. A. Cools A. R. (1990). Animal models with construct validity for schizophrenia. Behav. Pharmacol. 1, 469–490 10.1097/00008877-199000160-00001 11175433

[B22] Elliott R. McKenna P. J. Robbins T. W. Sahakian B. J. (1998). Specific neuropsychological deficits in schizophrenic patients with preserved intellectual function. Cogn. Neuropsychiatry 3, 45–70 10.1080/13546809839624214567169

[B23] Fenton A. A. (2009). “Neural coordination and psychotic disorganization,” in Information Processing by Neuronal Populations, eds Holscher C. Munk M. H. (London: Cambridge University Press), 387–408

[B24] Fenton A. A. Bureš J. (1993). Place navigation in rats with unilateral TTX inactivation of the dorsal hippocampus: place but not procedural learning can be lateralised to one hippocampus. Behav. Neurosci. 107, 552–564 10.1037/0735-7044.107.4.552 8397860

[B25] Fenton A. A. Kenney J. Kao H.-Y. (2006). Phencyclidine impairs cognition if and only if it co-activates initially independently active neurons. *Soc. Biol. Psychiatry* 59, 85S

[B26] Fenton A. A. Lytton W. W. Barry J. M. Lenck-Santini P. P. Zinyuk L. E. Kubík S. (2010). Attention-like modulation of hippocampus place cell discharge. J. Neurosci. 30, 4613–4625 10.1523/JNEUROSCI.5576-09.2010 20357112 PMC2858227

[B27] Fenton A. A. Wesierska M. Kaminsky Y. Bureš J. (1998). Both here and there: simultaneous expression of autonomous spatial memories in rats. Proc. Natl. Acad. Sci. U.S.A. 95, 11493–11498 10.1073/pnas.95.19.11493 9736765 PMC21671

[B28] Gilbert P. E. Kesner R. P. Lee I. (2001). Dissociating hippocampal subregions: double dissociation between dentate gyrus and CA1. Hippocampus 11, 626–636 10.1002/hipo.1077 11811656

[B29] Gold J. M. Waltz J. A. Matveeva T. M. Kasanova Z. Strauss G. P. Herbener E. S. (2012). Negative symptoms and the failure to represent the expected reward value of actions: behavioral and computational modeling evidence. Arch. Gen. Psychiatry 69, 129–138 10.1001/archgenpsychiatry.2011.1269 22310503 PMC4406055

[B30] Green M. F. (1996). What are the functional consequences of neurocognitive deficits in schizophrenia? Am. J. Psychiatry 153, 321–330 8610818 10.1176/ajp.153.3.321

[B31] Green M. F. Kern R. S. Heaton R. K. (2004). Longitudinal studies of cognition and functional outcome in schizophrenia: implications for MATRICS. Schizophr. Res. 72, 41–51 10.1016/j.schres.2004.09.009 15531406

[B32] Green M. F. Nuechterlein K. H. (1999). Should schizophrenia be treated as a neurocognitive disorder? Schizhopr. Bull. 25, 309–319 10.1093/oxfordjournals.schbul.a033380 10416733

[B33] Grunze H. C. Rainnie D. G. Hasselmo M. E. Barkai E. Hearn E. F. McCarley R. W. (1996). NMDA-dependent modulation of CA1 local circuit inhibition. J. Neurosci. 16, 2034–2043 8604048 10.1523/JNEUROSCI.16-06-02034.1996 PMC6578507

[B34] Guzowski J. F. Knierim J. J. Moser E. I. (2004). Ensemble dynamics of hippocampal regions CA1 and CA3. Neuron 44, 581–584 10.1016/j.neuron.2004.11.003 15541306

[B35] Guzowski J. F. Lyford G. L. Stevenson G. D. Houston F. P. McGaugh J. L. Worley P. F. (2000). Inhibition of activity-dependent arc protein expression in the rat hippocampus impairs the maintenance of long-term potentiation and the consolidation of long-term memory. J. Neurosci. 20, 3993–4001 10818134 10.1523/JNEUROSCI.20-11-03993.2000 PMC6772617

[B36] Guzowski J. F. McNaughton B. L. Barnes C. A. Worley P. F. (1999). Environment-specific expression of the immediate-early gene *Arc* in hippocampal neuronal ensembles. Nat. Neurosci. 2, 1120–1124 10.1038/16046 10570490

[B37] Harrison P. J. (2004). The hippocampus in schizophrenia: a review of the neuropathological evidence and its pathophysiological implications. Psychopharmacology (Berl.) 174, 151–162 10.1007/s00213-003-1761-y 15205886

[B38] Harvey P. D. Keefe R. S. (2001). Studies of cognitive change in patients with schizophrenia following novel antipsychotic treatment. Am. J. Psychiatry 158, 176–184 10.1176/appi.ajp.158.2.176 11156796

[B39] Hemsley D. R. (2005). The schizophrenic experience: taken out of context? Schizhopr. Bull. 31, 43–53 10.1093/schbul/sbi003 15888424

[B40] Hill S. K. Bishop J. R. Palumbo D. Sweeney J. A. (2010). Effect of second-generation antipsychotics on cognition: current issues and future challenges. Expert Rev. Neurother. 10, 43–57 10.1586/ern.09.143 20021320 PMC2879261

[B41] Jackson M. E. Homayoun H. Moghaddam B. (2004). NMDA receptor hypofunction produces concomitant firing rate potentiation and burst activity reduction in the prefrontal cortex. Proc. Natl. Acad. Sci. U.S.A. 101, 8467–8472 10.1073/pnas.0308455101 15159546 PMC420417

[B42] Ježek K. Henriksen E. J. Treves A. Moser E. I. Moser M.-B. (2011). Theta-paced flickering between place-cell maps in the hippocampus. Nature 478, 246–249 10.1038/nature10439 21964339

[B43] Keefe R. S. E. Poe M. Walker T. M. Kang J. W. Harvey P. D. (2006). The Schizophrenia Cognition Rating Scale: an interview-based assessment and its relationship to cognition, real-world functioning, and functional capacity. Am. J. Psychiatry 163, 426–432 10.1176/appi.ajp.163.3.426 16513863

[B44] Kelemen E. Fenton A. A. (2010). Dynamic grouping of hippocampal neural activity during cognitive control of two spatial frames. PLoS Biol. 8:e1000403 10.1371/journal.pbio.1000403 20585373 PMC2889929

[B45] Kelemen E. Fenton A. A. (2013). Key features of human episodic recollection in the cross-episode retrieval of rat hippocampus representations of space. PLoS Biol. 11:e1001607 10.1371/journal.pbio.1001607 23874154 PMC3712911

[B46] Kerwin R. Patel S. Meldrum B. (1990). Quantitative autoradiographic analysis of glutamate binding sites in the hippocampal formation in normal and schizophrenic brain post mortem. Neuroscience 39, 25–32 10.1016/0306-4522(90)90219-T 1982465

[B47] Kobayashi K. (2009). Targeting the hippocampal mossy fiber synapse for the treatment of psychiatric disorders. Mol. Neurobiol. 39, 24–36 10.1007/s12035-008-8049-5 19130314

[B48] Kolomeets N. S. Orlovskaya D. D. Rachmanova V. I. Uranova N. A. (2005). Ultrastructural alterations in hippocampal mossy fiber synapses in schizophrenia: a postmortem morphometric study. Synapse 57, 47–55 10.1002/syn.20153 15858835

[B49] Kolomeets N. S. Orlovskaya D. D. Uranova N. A. (2007). Decreased numerical density of CA3 hippocampal mossy fiber synapses in schizophrenia. Synapse 61, 615–621 10.1002/syn.20405 17476682

[B50] Kubik Š Fenton A. A. (2005). Behavioral evidence that segregation and representation are dissociable hippocampal functions. J. Neurosci. 25, 9205–9212 10.1523/JNEUROSCI.1707-05.2005 16207880 PMC6725773

[B51] Kubik Š Miyashita T. Guzowski J. F. (2007). Using immediate-early genes to map hippocampal subregional functions. Learn. Mem. 14, 758–770 10.1101/lm.698107 18007019

[B52] Kubik Š Miyashita T. Kubik-Zahorodna A. Guzowski J. F. (2012). Loss of activity-dependent *Arc* gene expression in the retrosplenial cortex after hippocampal inactivation: interaction in a higher-order memory circuit. Neurobiol. Learn. Mem. 97, 124–131 10.1016/j.nlm.2011.10.004 22100445

[B53] Ledoux A. A. Phillips J. L. Labelle A. Smith A. Bohbot V. D. Boyer P. (2013). Decreased fMRI activity in the hippocampus of patients with schizophrenia compared to healthy control participants, tested on a wayfinding task in a virtual town. Psychiatry Res. 211, 47–56 10.1016/j.pscychresns.2012.10.005 23352276

[B54] Lee H. Dvorak D. Kao H.-Y. Duffy A. M. Scharfman H. E. Fenton A. A. (2012). Early cognitive experience prevents adult deficits in a neurodevelopmental schizophrenia model. Neuron 75, 714–724 10.1016/j.neuron.2012.06.016 22920261 PMC3437240

[B55] Lee I. Yoganarasimha D. Rao G. Knierim J. J. (2004). Comparison of population coherence of place cells in hippocampal subfields CA1 and CA3. Nature 430, 456–459 10.1038/nature02739 15229614

[B56] Leutgeb J. K. Leutgeb S. Moser M. B. Moser E. I. (2007). Pattern separation in the dentate gyrus and CA3 of the hippocampus. Science 315, 961–966 10.1126/science.1135801 17303747

[B57] Leutgeb S. Leutgeb J. K. Treves A. Moser M.-B. Moser E. I. (2004). Distinct ensemble codes in hippocampal areas CA3 and CA1. Science 305, 1295–1298 10.1126/science.1100265 15272123

[B58] Lewis D. A. Hashimoto T. Volk D. W. (2005). Cortical inhibitory neurons and schizophrenia. Nat. Rev. Neurosci. 6, 312–324 10.1038/nrn1648 15803162

[B59] Li Q. Clark S. Lewis D. V. Wilson W. A. (2002). NMDA receptor antagonists disinhibit rat posterior cingulate and retrosplenial cortices: a potential mechanism of neurotoxicity. J. Neurosci. 22, 3070–3080 11943810 10.1523/JNEUROSCI.22-08-03070.2002 PMC6757533

[B60] Link W. Konietzko U. Kauselmann G. Krug M. Schwanke B. Frey U. (1995). Somatodendritic expression of an immediate early gene is regulated by synaptic activity. Proc. Natl. Acad. Sci. U.S.A. 92, 5734–5738 10.1073/pnas.92.12.5734 7777577 PMC41771

[B61] Lisman J. E. Coyle J. T. Green R. W. Javitt D. C. Benes F. M. Heckers S. (2008). Circuit-based framework for understanding neurotransmitter and risk gene interactions in schizophrenia. Trends Neurosci. 31, 234–242 10.1016/j.tins.2008.02.005 18395805 PMC2680493

[B62] Lobellova V. Entlerova M. Svojanovska B. Hatalova H. Prokopova I. Petrasek T. (2013). Two learning tasks provide evidence for disrupted behavioural flexibility in an animal model of schizophrenia-like behaviour induced by acute MK-801: a dose-response study. Behav. Brain Res. 246, 55–62 10.1016/j.bbr.2013.03.006 23499708

[B63] Lyford G. L. Yamagata K. Kaufmann W. E. Barnes C. A. Sanders L. K. Copeland N. G. (1995). Arc, a growth factor and activity-regulated gene, encodes a novel cytoskeleton-associated protein that is enriched in neuronal dendrites. Neuron 14, 433–445 10.1016/0896-6273(95)90299-6 7857651

[B64] Manschreck T. C. Merrill A. M. Jabbar G. Chun J. Delisi L. E. (2012). Frequency of normative word associations in the speech of individuals at familial high-risk for schizophrenia. Schizophr. Res. 140, 99–103 10.1016/j.schres.2012.06.034 22819779 PMC3732737

[B65] Martin P. Waters N. Waters S. Carlsson A. Carlsson M. L. (1997). MK-801-induced hyperlocomotion: differential effects of M100907, SDZ PSD 958 and raclopride. Eur. J. Pharmacol. 335, 107–116 10.1016/S0014-2999(97)01188-6 9369362

[B66] McHugh T. J. Jones M. W. Quinn J. J. Balthasar N. Coppari R. Elmquist J. K. (2007). Dentate gyrus NMDA receptors mediate rapid pattern separation in the hippocampal network. Science 317, 94–99 10.1126/science.1140263 17556551

[B67] Miller R. (1989). Hyperactivity of associations in psychosis. Aust. N. Z. J. Psychiatry 23, 241–248 10.3109/00048678909062141 2673199

[B68] Mishara A. L. Goldberg T. E. (2004). A meta-analysis and critical review of the effects of conventional neuroleptic treatment on cognition in schizophrenia: opening a closed book. Biol. Psychiatry 55, 1013–1022 10.1016/j.biopsych.2004.01.027 15121486

[B69] Miyashita T. Kubík Š Haghighi N. Steward O. Guzowski J. F. (2009). Rapid activation of plasticity-associated gene transcription in hippocampal neurons provides a mechanism for encoding of one-trial experience. J. Neurosci. 29, 898–906 10.1523/JNEUROSCI.4588-08.2009 19176799 PMC2749324

[B70] Moscovitch M. Nadel L. Winocur G. Gilboa A. Rosenbaum R. S. (2006). The cognitive neuroscience of remote episodic, semantic and spatial memory. Curr. Opin. Neurobiol. 16, 179–190 10.1016/j.conb.2006.03.013 16564688

[B71] Moser E. I. Moser M. B. (1998). Distributed encoding and retrieval of spatial memory in the hippocampus. J. Neurosci. 18, 7535–7542 9736671 10.1523/JNEUROSCI.18-18-07535.1998 PMC6793256

[B72] Newcomer J. W. Krystal J. H. (2001). NMDA receptor regulation of memory and behavior in humans. Hippocampus 11, 529–542 10.1002/hipo.1069 11732706

[B73] Nikiforuk A. Popik P. (2012). Effects of quetiapine and sertindole on subchronic ketamine-induced deficits in attentional set-shifting in rats. Psychopharmacology (Berl.) 220, 65–74 10.1007/s00213-011-2487-x 21918808 PMC3276756

[B74] Nowakowski C. Kaufmann W. A. Adlassnig C. Maier H. Salimi K. Jellinger K. A. (2002). Reduction of chromogranin B-like immunoreactivity in distinct subregions of the hippocampus from individuals with schizophrenia. Schizophr. Res. 58, 43–53 10.1016/S0920-9964(01)00389-9 12363389

[B75] Olypher A. V. Klement D. Fenton A. A. (2006). Cognitive disorganization in hippocampus: physiological model of the disorganization in psychosis. J. Neurosci. 26, 158–168 10.1523/JNEUROSCI.2064-05.2006 16399683 PMC6674308

[B76] Phillips W. A. Silverstein S. M. (2003). Convergence of biological and psychological perspectives on cognitive coordination in schizophrenia. Behav. Brain Sci. 26, 65–82 10.1017/S0140525X03000025 14598440

[B77] Plath N. Ohana O. Dammermann B. Errington M. L. Schmitz D. Gross C. (2006). *Arc/Arg3.1* is essential for the consolidation of synaptic plasticity and memories. Neuron 52, 437–444 10.1016/j.neuron.2006.08.024 17088210

[B78] Powel S. B. Geyer M. A. (2007). Overview of animal models of schizophrenia. Curr. Protoc. Neurosci. 39, 9.24.1–9.24.20 10.1002/0471142301.ns0924s39 18428667

[B79] Ragland J. D. Yoon J. Minzenberg M. J. Carter C. S. (2007). Neuroimaging of cognitive disability in schizophrenia: search for a pathophysiological mechanism. Int. Rev. Psychiatry 19, 417–427 10.1080/09540260701486365 17671874 PMC4332575

[B80] Ranganath C. Minzenberg M. J. Ragland J. D. (2008). The cognitive neuroscience of memory function and dysfunction in schizophrenia. Biol. Psychiatry 64, 18–25 10.1016/j.biopsych.2008.04.011 18495087 PMC2474810

[B81] Reichenberg A. Caspi A. Harrington H. Houts R. Keefe R. S. E. Murray R. M. (2010). Static and dynamic cognitive deficits in childhood preceding adult schizophrenia: a 30-year study. Am. J. Psychiatry 167, 160–169 10.1176/appi.ajp.2009.09040574 20048021 PMC3552325

[B82] Romón T. Mengod G. Adell A. (2011). Expression of parvalbumin and glutamic acid decarboxylase-67 after acute administration of MK-801. Implications for the NMDA hypofunction model of schizophrenia. Psychopharmacology (Berl.) 217, 231–238 10.1007/s00213-011-2268-6 21465242

[B83] Rorabacher D. B. (1991). Statistical treatment for rejection of deviant values: critical values of Dixon’s “Q” parameter and related subrange ratios at the 95% confidence level. Anal. Chem. 63, 139–146 10.1021/ac00002a010

[B84] Rosenheck R. CATIE Study Investigators Group Leslie D. Keefe R. McEvoy J. Swartz M. (2006). Barriers to employment for people with schizophrenia. Am. J. Psychiatry 163, 411–417 10.1176/appi.ajp.163.3.411 16513861

[B85] Rung J. P. Carlsson A. Rydén Markinhuhta K. Carlsson M. L. (2005). (+)-MK-801 induced social withdrawal in rats; a model for negative symptoms of schizophrenia. Prog. Neuropsychopharmacol. Biol. Psychiatry 29, 827–832 10.1016/j.pnpbp.2005.03.004 15916843

[B86] Schneider F. Gur R. C. Koch K. Backes V. Amunts K. Shah N. J. (2006). Impairment in the specificity of emotion processing in schizophrenia. Am. J. Psychiatry 163, 442–447 10.1176/appi.ajp.163.3.442 16513865

[B87] Silverstein S. M. Kovacs I. Corry R. Valone C. (2000). Perceptual organization, the disorganization syndrome, and context processing in chronic schizophrenia. Schizophr. Res. 43, 11–20 10.1016/S0920-9964(99)00180-2 10828411

[B88] Sørensen H. J. Mortensen E. L. Schiffman J. Reinisch J. M. Maeda J. Mednick S. A. (2010). Early developmental milestones and risk of schizophrenia: a 45-year follow-up of the Copenhagen Perinatal Cohort. Schizophr. Res. 118, 41–47 10.1016/j.schres.2010.01.029 20181463 PMC3676634

[B89] Steward O. Worley P. F. (2001). Selective targeting of newly synthesized Arc mRNA to active synapses requires NMDA receptor activation. Neuron 30, 227–240 10.1016/S0896-6273(01)00275-6 11343657

[B90] Stuchlík A. Fenton A. A. Bureš J. (2001). Substratal idiothetic navigation of rats is impaired by removal or devaluation of extramaze and intramaze cues. Proc. Natl. Acad. Sci. U.S.A. 98, 3537–3542 10.1073/pnas.051630498 11248113 PMC30688

[B91] Stuchlík A. Rezácová L. Valeš K. Bubeníková V. Kubík S. (2004). Application of a novel active allothetic place avoidance task (AAPA) in testing a pharmacological model of psychosis in rats: comparison with the Morris Water Maze. Neurosci. Lett. 366, 162–166 10.1016/j.neulet.2004.05.037 15276239

[B92] Stuchlík A. Valeš K. (2005). Systemic administration of MK-801, a non-competitive NMDA-receptor antagonist, elicits a behavioural deficit of rats in the active allothetic place avoidance (AAPA) task irrespectively of their intact spatial pretraining. Behav. Brain Res. 159, 163–171 10.1016/j.bbr.2004.10.013 15795010

[B93] Tamminga C. A. Southcott S. Sacco C. Wagner A. D. Ghose S. (2012). Glutamate dysfunction in hippocampus: relevance of dentate gyrus and CA3 signaling. Schizophr. Bull. 38, 927–935 10.1093/schbul/sbs062 22532703 PMC3446225

[B94] Tamminga C. A. Stan A. D. Wagner A. D. (2010). The hippocampal formation in schizophrenia. Am. J. Psychiatry 167, 1178–1193 10.1176/appi.ajp.2010.09081187 20810471

[B95] Tüscher O. Silbersweig D. Pan H. Smith T. Beutel M. Zonana J. (2005). Processing of environmental sounds in schizophrenic patients: disordered recognition and lack of semantic specificity. Schizophr. Res. 73, 291–295 10.1016/j.schres.2004.06.010 15653274

[B96] Uhlhaas P. J. Silverstein S. M. Phillips W. A. Lovell P. G. (2004). Evidence for impaired visual context processing in schizotypy with thought disorder. Schizophr. Res. 68, 249–260 10.1016/S0920-9964(03)00184-1 15099607

[B97] Valeš K. Bubeníková-Valešová V. Klement D. Stuchlík A. (2006). Analysis of sensitivity to MK-801 treatment in a novel active allothetic place avoidance task and in the working memory version of the Morris water maze reveals differences between Long-Evans and Wistar rats. Neurosci. Res. 55, 383–388 10.1016/j.neures.2006.04.007 16712995

[B98] van der Staay F. J. Rutten K. Erb C. Bloklande A. (2011). Effects of the cognition impairer MK-801 on learning and memory in mice and rats. Behav. Brain Res. 220, 215–229 10.1016/j.bbr.2011.01.052 21310186

[B99] Vazdarjanova A. Guzowski J. F. (2004). Differences in hippocampal neuronal population responses to modifications of an environmental context: evidence for distinct, yet complementary, functions of CA3 and CA1 ensembles. J. Neurosci. 24, 6489–6496 10.1523/JNEUROSCI.0350-04.2004 15269259 PMC6729865

[B100] Vazdarjanova A. McNaughton B. L. Barnes C. A. Worley P. F. Guzowski J. F. (2002). Experience-dependent coincident expression of the effector immediate-early genes *Arc* and *Homer 1a* in hippocampal and neocortical neuronal networks. J. Neurosci. 22, 10067–10071 12451105 10.1523/JNEUROSCI.22-23-10067.2002 PMC6758761

[B101] Vollenweider F. X. Leenders K. L. Scharfetter C. Antonini A. Maguire P. Missimer J. (1997). Metabolic hyperfrontality and psychopathology in the ketamine model of psychosis using positron emission tomography (PET) and [18F]fluorodeoxyglucose (FDG). Eur. Neuropsychopharmacol. 7, 9–24 10.1016/S0924-977X(96)00039-9 9088881

[B102] Weinberger D. R. (1999). Cell biology of the hippocampal formation in schizophrenia. Biol. Psychiatry 45, 395–402 10.1016/S0006-3223(98)00331-X 10071707

[B103] Weiss A. P. Schacter D. L. Goff D. C. Rauch S. L. Alpert N. M. Fischman A. J. (2003). Impaired hippocampal recruitment during normal modulation of memory performance in schizophrenia. Biol. Psychiatry 53, 48–55 10.1016/S0006-3223(02)01541-X 12513944

[B104] Wentura D. Moritz S. Frings C. (2008). Further evidence for “hyper-priming” in thought-disordered schizophrenic patients using repeated masked category priming. Schizophr. Res. 102, 69–75 10.1016/j.schres.2008.04.016 18511239

[B105] Wesierska M. Dockery C. Fenton A. A. (2005). Beyond memory, navigation and inhibition: behavioural evidence for hippocampus-dependent cognitive coordination in the rat. J. Neurosci. 25, 2413–2419 10.1523/JNEUROSCI.3962-04.2005 15745968 PMC6726107

[B106] Westerhausen R. Kompus K. Hugdahl K. (2011). Impaired cognitive inhibition in schizophrenia: a meta-analysis of the Stroop interference effect. Schizophr. Res. 133, 172–181 10.1016/j.schres.2011.08.025 21937199

[B107] Yamasaki N. Maekawa M. Kobayashi K. Kajii Y. Maeda J. Soma M. (2008). Alpha-CaMKII deficiency causes immature dentate gyrus, a novel candidate endophenotype of psychiatric disorders. Mol. Brain 1, 6 10.1186/1756-6606-1-6 18803808 PMC2562999

[B108] Zemanova A. Stankova A. Lobellova V. Svoboda J. Vales K. Vlcek K. (2013). Visuospatial working memory is impaired in an animal model of schizophrenia induced by acute MK-801: an effect of pretraining. Pharmacol. Biochem. Behav. 106, 117–123 10.1016/j.pbb.2013.03.014 23558085

[B109] Zinyuk L. Kubík S. Kaminsky Y. Fenton A. A. Bureš J. (2000). Understanding hippocampal activity by using purposeful behavior: place navigation induces place cell discharge in both task-relevant and task irrelevant spatial reference frames. Proc. Natl. Acad. Sci. U.S.A. 97, 3771–3776 10.1073/pnas.97.7.3771 10716713 PMC16315

